# Empirically derived dietary patterns are associated with major adverse cardiovascular events, all-cause mortality, and congestive cardiac failure in older men: The Concord Health and Ageing in Men Project

**DOI:** 10.1016/j.jnha.2023.100020

**Published:** 2024-01-01

**Authors:** Rebecca Luong, Rosilene Ribeiro, Vasi Naganathan, Fiona Blyth, Louise M Waite, David J Handelsman, David G Le Couteur, Markus J Seibel, Vasant Hirani

**Affiliations:** a Nutrition and Dietetics Group, Sydney Nursing School, Faculty of Medicine and Health, The University of Sydney, NSW, Australia; b ARC Centre of Excellence in Population Ageing Research (CEPAR), The University of Sydney, NSW, Australia; c Charles Perkins Centre, The University of Sydney, NSW, Australia; d School of Life and Environmental Sciences, Faculty of Science, The University of Sydney, NSW, Australia; e Centre for Education and Research on Ageing, Concord Hospital, The University of Sydney, Concord, NSW, Australia; f Concord Clinical School, Faculty of Medicine and Health, The University of Sydney, Concord, NSW, Australia; g School of Public Health, The University of Sydney, NSW, Australia; h ANZAC Research Institute, The University of Sydney, Concord, NSW, Australia; i Andrology Department, Concord Hospital, Concord, NSW, Australia

**Keywords:** Diet, Food, Older men, Mortality, Heart failure, Coronary artery disease

## Abstract

**Background:**

Diet is associated with major adverse cardiovascular events (MACE).

**Objective:**

We evaluated the associations between empirically derived dietary patterns and MACE.

**Design:**

Prospective cohort study.

**Setting:**

The Concord Health and Ageing in Men Project, Sydney, Australia.

**Participants:**

539 community-dwelling older Australian men aged 75 years and older.

**Methods:**

Men underwent dietary assessment using a validated dietitian-administered diet history questionnaire. Cox regression analyses were conducted between MACE and the three dietary patterns identified from factor analysis. Five-point MACE comprised of all-cause mortality, myocardial infarction (MI), congestive cardiac failure (CCF), coronary revascularisation, and/or ischaemic stroke. Four-point MACE included the four endpoints of MI, CCF, coronary revascularisation, and/or ischaemic stroke, and excluded all-cause mortality.

**Results:**

At a median of 5.3 (IQR 4.6–6.3) years of follow-up, the incidences were: five-point MACE 31.2% (n = 168); four-point MACE excluding all-cause mortality 17.8% (n = 96); all-cause mortality 20.1% (n = 111); CCF 11.3% (n = 61); MI 3.7% (n = 20); stroke 3.2% (n = 17); and coronary revascularisation 3.1% (n = 15). In fully adjusted analyses, compared to the bottom tertile, the middle tertile of ‘vegetables-legumes-seafood’ dietary pattern was associated with reduced five-point MACE (HR 0.67 [95% CI: 0.45, 0.99, P = .047]), and CCF (HR 0.31 [95% CI: 0.15, 0.65, P = .002]), whilst the middle tertile of ‘wholegrains-milk-other fruits’ dietary pattern was associated with increased five-point MACE (HR 1.78 [95% CI: 1.17, 2.70, P = .007]), four-point MACE (HR 1.92 [95% CI: 1.12, 3.30, P = .018]), and CCF (HR 2.33 [95% CI: 1.17, 4.65, P = .016]). For the ‘discretionary-starchy vegetables-processed meats’ dietary pattern, a higher score was associated with increased five-point MACE (HR 1.33 [95% CI: 1.09, 1.62, P = .004]), and all-cause mortality (HR 1.63 [95% CI: 1.26, 2.12, P < .001]), and compared to the bottom tertile, the top tertile was associated with increased all-cause mortality (HR 2.26 [95% CI: 1.27, 4.00, P = .005]).

**Conclusion:**

Older men may benefit from consuming a ‘vegetables-legumes-seafood’ dietary pattern rather than ‘discretionary-starchy vegetables-processed meats’ and ‘wholegrains-milk-other fruits’ dietary patterns for the prevention of MACE.

## 1 Introduction

Cardiovascular diseases (CVD) predict major adverse cardiovascular events (MACE) and are the leading causes of mortality worldwide [1,2]. Diet has protective and causal effects on health and there is emerging evidence on the different effects in older adults compared to their younger counterparts, whereby some research showed weaker associations between dietary patterns and CVD risk factors in adults aged 60 years and over [3,4].

Dietary patterns are composites of foods, nutrients, other dietary factors, and their consumption frequency, and is representative of synergistic effects on health rather than the single effects from individual dietary components [5]. Dietary patterns are derived from three main approaches: the *a posteriori* or data-driven approach, the *a priori* or hypothesis-driven approach, and the hybrid approach which combines the *a posteriori* and *a priori* approaches [6,7]. Although each approach has its advantages and limitations, the *a posteriori* approach, which involves empirically derived dietary patterns, offers notable benefits [7]. This approach is independent of existing knowledge, takes into account multiple dimensions, provides valuable insights into the interrelationships between food combinations and the habitual dietary patterns adopted by individuals [7].

The Concord Health and Ageing in Men Project (CHAMP) is a prospective cohort study of ageing in men. Men were the focus of the CHAMP study because epidemiological studies of ageing tended to include less men [8]. Men have a shorter life expectancy, and older men experience double the rates of CVD than older women [8,9]. Through the CHAMP cohort, it has been previously shown that *a priori* indices including the revised Dietary Guideline Index was cross-sectionally associated with improved cardiometabolic health parameters [10] and the pyramid-based Mediterranean diet score was longitudinally associated with reduced risks of developing MACE over 4.7 years in older men aged 75 years and over [11]. *A priori* indices based on selected aspects do not describe the overall dietary pattern [7]. Furthermore, there are limited longitudinal studies investigating the associations between *a posteriori* empirically derived dietary patterns and MACE, with a lack of sex-specific research and populations in the later stages of the life cycle [12]. Additionally, previous studies mostly involved CHD, CVD, and mortality as the outcome rather than MACE and the individual endpoints of MACE [[12], [13], [14]].

Therefore, the aim of the current study was to evaluate the associations between empirically derived dietary patterns with MACE, and individual endpoints of MACE, in older men aged 75 years and over.

## 2 Methods

### 2.1 Study participants

**Fig. 1:**
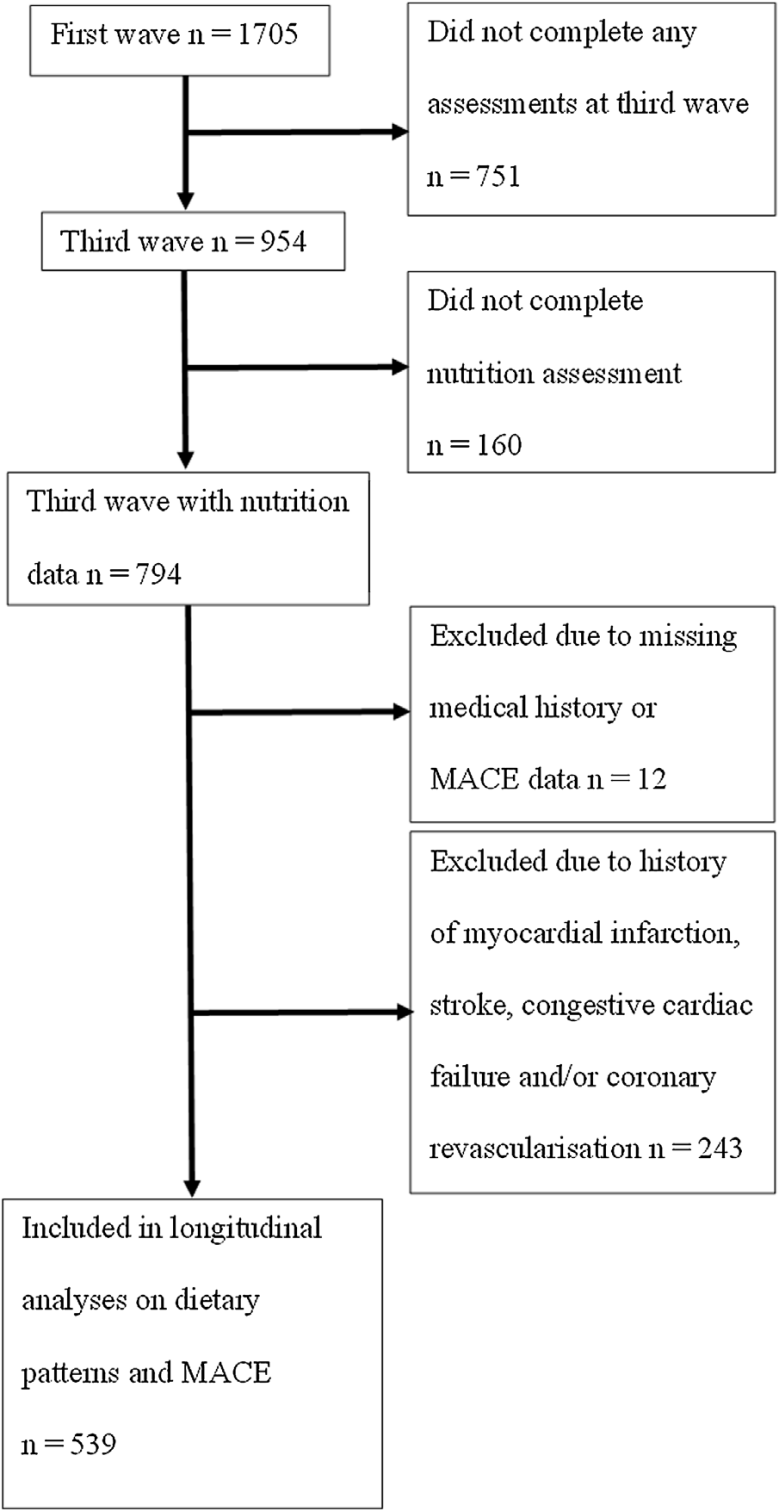
Flow diagram of participants included in analyses.

Participants were recruited from a defined geographic region of three Local Government Areas around Concord Hospital in Sydney (Burwood, Canada Bay and Strathfield) using the New South Wales electoral roll in which enrolment is compulsory in Australia. A total of 1705 men aged 70 years and over were recruited in the first wave of the CHAMP study (between January 2005 and June 2007) [8]. The third wave of CHAMP involved 954 men aged 75 years and over, of which 794 men participated in the dietary data collection (between August 2010 and August 2013, baseline nutrition) [10]. Of these men, 782 (98.4%) had both MACE and medical history data available. A total of 243 men had a history of myocardial infarction (MI), stroke, congestive cardiac failure (CCF), and/or coronary revascularisation identified from self-reported questionnaires at third wave and data linkage of MACE from first wave up to third wave of CHAMP, and were excluded. Thus, a total of 539 men were included in the prospective analyses on associations between empirically derived dietary patterns and MACE. Flowchart of participants’ inclusion in analyses is shown in Fig. 1. The CHAMP study was conducted according to the guidelines laid down in the Declaration of Helsinki and all procedures involving human subjects were approved by the Concord Hospital Human Research Ethics Committee (HREC/14/CRGH/17). Written informed consent was obtained from all participants.

### 2.2 Dietary intake

Dietary data was collected using a validated dietitian-administered diet history questionnaire at baseline. Participants were asked about their usual dietary intake in the previous 3 months and food models, photographs, and household measures were used to assist with estimation of amounts consumed [15,16]. A food checklist was included, and relatives, carers, and/or family members of participants were encouraged to attend the interview to aid memory recall [16]. The validity of this diet history questionnaire by comparison with a prospective 4-day weighed food record which does not rely on participants’ memory was previously reported in a subgroup of 56 CHAMP men [16]. The mean differences in dietary intakes between the two methods were generally less than 20% [16].

Dietary data was initially analysed using FoodWorks 7 Professional for Windows (Xyris Software (Brisbane, Australia) Pty Ltd) that utilised The Australian Food, Supplement and Nutrient Database 2007 (AUSNUT 2007) [10]. A matching file was used to update the dietary data to The Australian Food, Supplement and Nutrient Database 2011–2013 (AUSNUT 2011–2013) and entries were converted to nutrients, foods, and food groups through FoodWorks [17]. A serve of each food group was based on the Australian Guide to Healthy Eating [18,19]. Discretionary foods are not necessary to provide the nutrients the body needs, are generally high in saturated fats, sugars, salt and/or alcohol, and thus are energy-dense and nutrient-poor according to the Australian Dietary Guidelines [20]. Due to the different classification of sugar, solid fat, and alcohol as food groups in FoodWorks, a serve of discretionary was defined as 4.2 g (1 teaspoon) sugar, 4.8 g (1 teaspoon) solid fat equivalents, or 10 g alcohol (1 standard drink) [10,19].

Dietary pattern exposure was defined as dietary intakes at baseline nutrition. The 23 food groups include: (i) refined grains; (ii) wholegrains; (iii) citrus, melons and berries; (iv) other fruit; (v) fruit juice; (vi) dark green vegetables; (vii) red orange vegetables (viii) starchy vegetables; (ix) other vegetables; (x) legumes; (xi) red meats; (xii) poultry; (xiii) eggs; (xiv) processed meats; (xv) organ meats; (xvi) seafood; (xvii) nuts and seeds; (xviii) soy products; (xix) milk (xx) cheese; (xxi) yoghurt; (xxii) milk alternatives; (xxiii) discretionary. Other vegetables are vegetables that are not dark green, red orange, or starchy. Other fruits are fruits other than citrus, melons and berries, or fruit juice.

### 2.3 MACE measurement

MACE outcomes are a composite index. Five-point MACE is comprised of all-cause mortality, MI, CCF, coronary revascularisation, and/or ischaemic stroke. Four-point MACE includes the four endpoints of MI, CCF, coronary revascularisation, and/or ischaemic stroke, and excludes all-cause mortality. Individual endpoints of MACE were censored on 31 December 2017 (7.4 years following nutrition assessment).

MACE data were obtained through data linkage undertaken by the NSW Centre for Health Record Linkage (CHeReL) from three population databases: New South Wales Admitted Patient Data Collection Registry; Births, Deaths and Marriages Registry; and Australian Bureau of Statistics Mortality Data using International Classification of Diseases coding (ICD-10) for the following: MI (codes: I21.0–I21.4, I21.9, I22.0–I22.1, I22.9); ischaemic stroke (codes: I63.0–I63.6, I63.8–I63.9); CCF (codes: I50.0–I50.1, I50.9). The Australian Classification of Health Interventions codes were used to identify participants who underwent coronary revascularisation, including coronary artery angioplasty (codes: 38300-00, 38300-01, 38303-00, 38303-01, 35309-06, 35309-07, 90218-00 to 90218-03), or coronary artery bypass grafting (codes: 38497-00 to 38497-07, 38500-00 to 38500-03, 38503-00, 38503-01, 38503-04, 38503-05, 90201-00 to 90201-03). Data on cause-specific mortality was not available.

### 2.4 Other measurements

Data on anthropometry, socio-demographics, lifestyle, and health factors were collected through self-reported and interviewer-administered clinic questionnaires including biochemical analyses, physical measurements, and medication inventory. Height, weight, waist circumference, and hip circumference were measured following standardised protocols as previously described [10]. Body mass index (BMI) was calculated as kg/m^2^. Physical activity was assessed through Physical Activity Scale for the Elderly (PASE) [21].

Marital status was categorised into ‘married/de facto’ and ‘not married/divorced/separated/widowed/never married/other’. ‘Age Pension only’ referred to those who only received the Age Pension, whilst ‘other’ referred to those with other sources of income apart from the Age Pension including veteran pension, repatriation pension, superannuation, private income, business ownership, farm ownership, business partnership, wage, salary, and/or other. Country of birth was categorised into ‘Australia’, ‘Greece/Italy’, and ‘other’. Smoking status was categorised into ‘nonsmoker’, ‘ex-smoker’, and ‘current smoker’ based on self-reported smoking history.

Prescription and non-prescription medication used daily or almost daily were brought to the clinic visit and recorded. Participants were asked whether they had taken any other medications during the past month. Reported medications were used to determine the number of medications and were coded using the Iowa Drug Information Service drug code numbers. Supplement use including vitamins, minerals, and/or fish oil was categorised into ‘yes’ and ‘no’. Number of cardiovascular medications was based on reported use of anti-coagulants and antiplatelets (including aspirin), anti-hypertensive agents (beta-adrenergic blockers, hypotensive agents, and vasodilating agents), cardiovascular agents (cardiac agents and cardiac glycosides), antilipemic agents, and diuretics.

Self-rated health was obtained through response to the question, ‘compared to other people of your age, how would you rate your health?’, and data was categorised into ‘very poor/poor/fair’, and ‘good/excellent’. Cognitive function was assessed using the Mini-Mental State Examination (MMSE) [22], which has a score range from 0 to 30 (higher scores indicating better cognition). Participants with a score of less than 24 were categorised as ‘cognitively impaired’, which has a sensitivity of 0.85 and specificity of 0.90 in studies of older adults in community settings [23]. Anaemia was defined as haemoglobin levels <130 g/L [24]. The inflammatory biomarker included was cytokine interleukin-6 (IL-6). Serum creatinine (Scr) levels in μmol/L multiplied by 0.0113 to convert to mg/dL, were used to estimate glomerular filtration rate (eGFR). Depending on the Scr levels, we used the following Chronic Kidney Disease Epidemiology Collaboration (CKD-EPI) equations for men [25]: Scr ≤ 80 μmol/L (i.e. ≤0.9 mg/dL): eGFR = 141 × (Scr/0.9)^−0.411^ × (0.993)^Age^; and Scr > 80 μmol/L (i.e. 0.9 mg/dL): eGFR = 141 × (Scr/0.9)^−1.209^ × (0.993)^Age^. Chronic kidney disease (CKD) was defined as eGFR < 60 mL/min/1.73 m^2^. The number of comorbidities was determined by the sum of all conditions that participants reported including: diabetes, thyroid disease, osteoporosis, Paget’s disease, stroke, Parkinson’s disease, kidney stones, dementia, depression, epilepsy, hypertension, angina, intermittent claudication, chronic obstructive pulmonary disease, liver disease, renal disease, arthritis, gout, and cancer (excluding nonmelanoma skin cancers).

Frailty was defined using the Fried frailty phenotype criteria according to the Cardiovascular Health Study (CHS) for weakness and slowness [26], and adapted criteria for weight loss, exhaustion, and low activity [27]. Participants were classified as robust if they had none, pre-frail if they had one or two, and frail if they had three or more of the five frailty components.

### 2.5 Dietary pattern analysis

Factor analysis using orthogonal transformation, varimax rotation with the 23 food groups as variables was conducted for the extraction of the dietary pattern factors. Factors were retained based on an eigenvalue >1.5, a breakpoint in the scree plot, and the interpretability [28]. Satisfactory factor analysis was determined through the Kaiser–Meyer–Olkin measure (>0.5 is acceptable) and the Bartlett’s test (P < 0.05 as statistically significant) [28]. Loadings of each food group represented the correlation between the food groups and the factor. Major contributions were determined by an absolute value of factor loading >0.30 [29,30]. Positive loadings represented alignment to the dietary pattern and negative loadings indicated non-alignment to that pattern. The higher the factor score for each dietary pattern indicated greater conformity with that dietary pattern for that individual.

### 2.6 Statistical analysis

Statistical analysis was carried out using SPSS software version 25 (IBM Corp., Armonk, NY, USA) [31]. Normality tests (histogram, Q-Q plot, and Shapiro-Wilk test) conducted found that most data had a skewed distribution. Descriptive characteristics were expressed as median (interquartile range) (IQR) and as number of participants (percentage of participants). Participant characteristics and dietary intake at nutrition assessment according to dietary pattern scores were compared. For categorical data, chi-square tests were used to compare participant characteristics across tertiles. For numerical data, median tests, and Bonferroni correction for multiple tests were used to compare participant characteristics and dietary intake between each pair of tertiles.

The Cox regression method was used to determine the association between dietary pattern scores with five-point MACE, four-point MACE excluding all-cause mortality, and in subanalyses individual endpoints of MACE. Results are presented as hazard ratios (HRs) with 95% CI. Dietary pattern scores were analysed as both continuous and categorical variables (i.e. categorised into tertiles with bottom as the reference category). Pre-specified potential confounders considered for inclusion in the Cox regression models were socio-demographic and lifestyle factors (age, BMI, country of birth, marital status, source of income, smoking status, PASE, energy intake, and supplement use including vitamins, minerals, and/or fish oil), health (number of cardiovascular medications, MMSE score, frailty, diabetes, CKD, cancer, IL-6, and haemoglobin). Backward elimination with likelihood ratio tests was used to build models containing dietary pattern scores as the explanatory variable against the outcome. Covariates included in the multivariable model were: age, BMI, country of birth, source of income, marital status, smoking status, energy intake, supplement use including vitamins, minerals, and/or fish oil, haemoglobin, number of cardiovascular medications, frailty status, history of cancer, and CKD. The proportional hazards assumption was determined using the graphical method (‘log minus log’), and time interaction with each included variable was tested and none were significant. Interactions between covariates in the finally adjusted model were tested and none were significant.

In subanalyses, frailty status could not be entered into adjusted models assessing associations between dietary pattern scores and coronary revascularisation due to no participants experiencing an event in a category. The third factor ‘discretionary-starchy vegetables-processed meats’ dietary pattern score as categorical tertiles and their associations with stroke could not be computed as no events occurred in one of the tertiles.

In subgroup analyses by age, 75–84 years and >85 years, not all covariates could be entered into adjusted models assessing associations between dietary pattern scores and coronary revascularisation, myocardial infarction, and stroke due to small numbers and/or no participants experiencing an event in a category. The first factor ‘vegetables-legumes-seafood’ dietary pattern score as categorical tertiles and their associations with stroke for older men aged 75–84 years could not be computed as no events occurred in one of the tertiles. The first factor ‘vegetables-legumes-seafood’ dietary patter score and second factor ‘wholegrains-milk-other fruits’ dietary pattern score as categorical tertiles and their associations with coronary revascularisation for older men aged >85 years could not be computed as no events occurred in one of the tertiles. The third factor ‘discretionary-starchy vegetables-processed meats’ dietary pattern score as categorical tertiles and their associations with stroke for older men aged 75–84 years and >85 years could not be computed as no events occurred in one of the tertiles.

Survival analysis plots of event-free survival were generated with cumulative event rates as a function of time and stratified by tertiles of dietary pattern scores for unadjusted and adjusted analyses.

## 3 Results

### 3.1 Dietary patterns

**Fig. 2:**
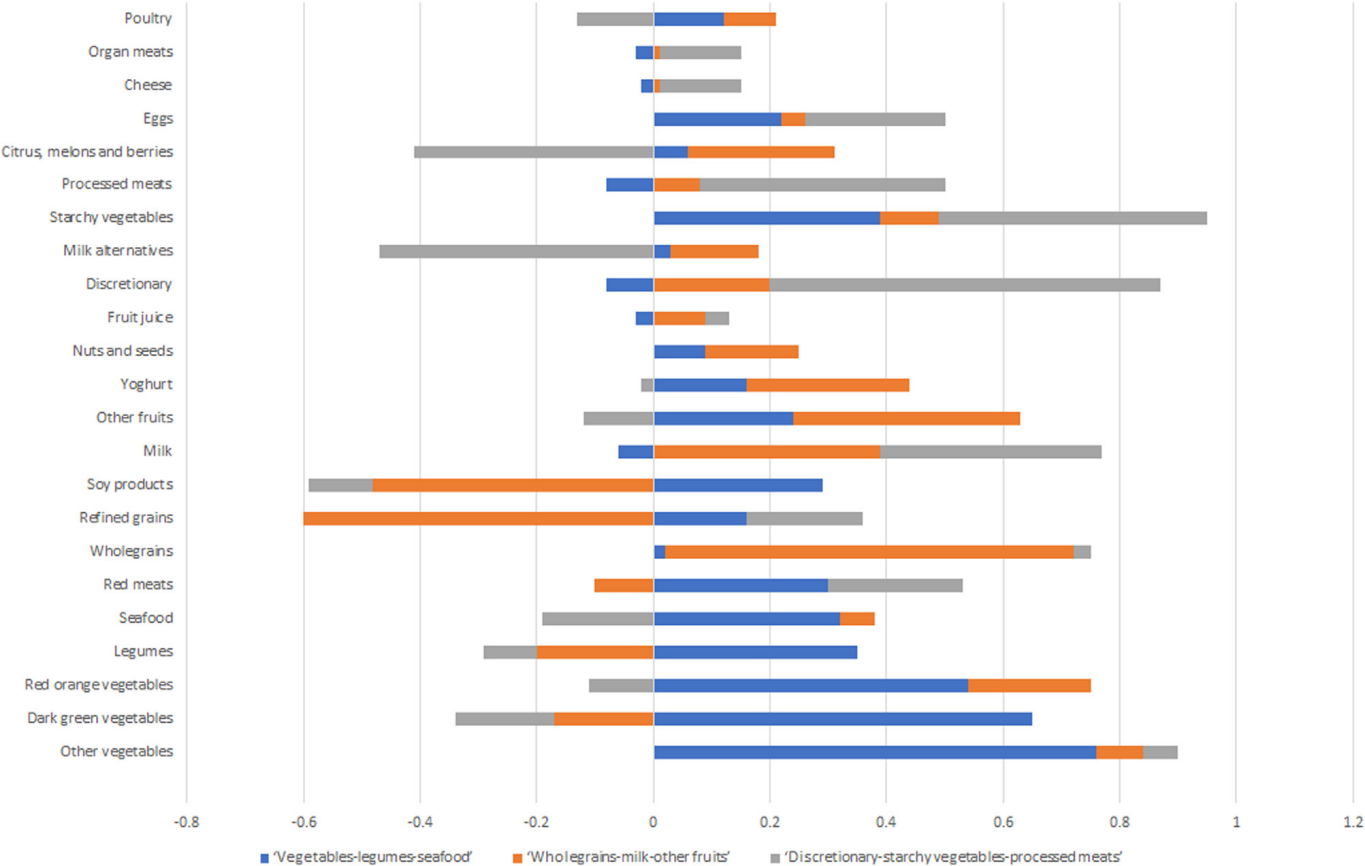
Factor loadings with varimax rotation to determine the association between 23 food groups and factors representing dietary patterns among older Australian men (n = 539). Factor loadings with absolute value >0.30 and <-0.30 indicate major contributing food groups and non-contributing food groups for each dietary pattern respectively.

Factor analysis identified three main dietary patterns for the population in the present study (scree plot shown in Supplementary Fig. 1). Food groups and factor loadings for each dietary pattern are presented in Fig. 2 (absolute factor loadings, % variance explained, and eigenvalues are shown in Supplementary Table 1). Dietary patterns were named according to major contributing food groups with high factor loadings: ‘vegetables-legumes-seafood’, ‘wholegrains-milk-other fruits’, and ‘discretionary-starchy vegetables-processed meats’. These dietary patterns respectively accounted for 9.13%, 7.73% and 7.07% of the variance in food intakes, explaining a total of 23.9% of the variability.

### 3.2 Participant characteristics

Table 1 presents the participant characteristics according to tertiles of dietary pattern scores. A total of 539 men without a history of MI, stroke, CCF, and/or coronary revascularisation had dietary intake and MACE data available at nutrition assessment. Participants’ median age was 80.0 (IQR 77.0−83.0) years and BMI was 27.3 (IQR 25.0−30.1) kg/m^2^. Characteristics that differed between the dietary pattern tertiles included BMI, hip circumference, marital status, source of income, country of birth, smoking status, number of cardiovascular medications, haemoglobin, and cognitive impairment. Supplementary Table 2 presents dietary intake according to tertiles of dietary pattern scores. As expected food group intakes and thus energy and nutrient intakes were also different between the dietary pattern tertiles. Soy products, yoghurt, fruit juice, milk alternatives, and organ meats were reportedly minimally consumed by all participants.

**Table 1:** Participant characteristics (median and interquartile range; percentages and number of participants) according to dietary pattern score tertiles (n = 539).

		Factor 1: ‘Vegetables-legumes-seafood’				Factor 2: ‘Wholegrains-milk-other fruits’				Factor 3: ‘Discretionary-starchy vegetables-processed meats’			
Variables	All	Bottom	Middle	Top	P value^1^	Bottom	Middle	Top	P value^1^	Bottom	Middle	Top	P value^1^
	n = 539	n = 180	n = 180	n = 179		n = 180	n = 180	n = 179		n = 180	n = 180	n = 179	
Age (years)	80.0 (77.0−83.0)	80.0 (78.0−84.0)	80.0 (77.0−83.0)	79.0 (77.0−83.0)	.17	80.0 (77.0−83.0)	80.0 (77.0−84.0)	80.0 (77.0−84.0)	.34	80.0 (77.0−83.0)	80.0 (77.0−83.0)	80.0 (77.0−84.0)	.51
BMI (kg/m^2^) (n = 533)	27.3 (25.0−30.1)	27.3 (25.2−30.0)^a,b^	28.0 (25.5−30.9)^a,c^	26.5 (24.5−29.1)^b,c^	.22^a^	27.5 (25.2−30.5)^a,b^	27.9 (25.7−30.8)^a,c^	26.6 (24.2−28.9)^b,c^	1.00^a^	27.2 (25.3−30.3)	27.2 (24.9−30.0)	27.5 (24.9−30.3)	.80
					.13^b^				.13^b^				
					.013^c^				.006^c^				
Waist circumference (cm) (n = 535)	101.3 (94.2−107.8)	102.2 (96.2−107.8)	102.6 (95.0−110.0)	99.9 (92.8−106.1)	.038†	100.6 (94.3−107.6)	102.8 (96.2−110.2)	99.8 (93.4−105.6)	.058	99.6 (93.6−107.6)	101.9 (94.4−107.2)	102.1 (95.7−109.8)	.13
Hip circumference (cm) (n = 535)	101.9 (97.5−107.6)	103.0 (98.0−108.0)^a,b^	102.3 (98.3−109.0)^a,c^	100.1 (96.6−106.0)^b,c^	1.00^a^	101.6 (97.0−107.5)	102.6 (98.4−108.7)	101.6 (97.0−107.0)	.64	100.8 (96.6−106.8)	101.9 (98.0−107.5)	102.5 (97.9−108.2)	.41
					.033^b^								
					0.12^c^								
Marital status (n = 536)					.012				.21				.26
Married/de facto	418 (78.0)	127 (70.9)	150 (83.8)	141 (79.2)		148 (82.2)	138 (77.1)	132 (74.6)		146 (82.0)	135 (75.0)	137 (77.0)	
Not married/divorced/separated/widowed/never married/other	118 (22.0)	52 (29.1)	29 (16.2)	37 (20.8)		32 (17.8)	41 (22.9)	45 (25.4)		32 (18.0)	45 (25.0)	41 (23.0)	
Source of income (n = 538)					.26				<.001				.26
Age Pension only	213 (39.6)	79 (44.1)	70 (38.9)	64 (35.8)		101 (56.1)	64 (35.6)	48 (27.0)		102 (56.7)	117 (65.0)	106 (59.6)	
Other	325 (60.4)	100 (55.9)	110 (61.1)	115 (64.2)		79 (43.9)	116 (64.4)	130 (73.0)		78 (43.3)	63 (35.0)	72 (40.4)	
Country of birth					.075				<.001				<.001
Australia	279 (51.8)	100 (55.6)	96 (53.3)	83 (46.4)		58 (32.2)	101 (56.1)	120 (67.0)		74 (41.1)	94 (52.2)	111 (62.0)	
Greece/Italy	131 (24.3)	36 (20.0)	51 (28.3)	44 (24.6)		77 (42.8)	39 (21.7)	15 (8.4)		68 (37.8)	33 (18.3)	30 (16.8)	
Other	129 (23.9)	44 (24.4)	33 (18.3)	52 (29.1)		45 (25.0)	40 (22.2)	44 (24.6)		38 (21.1)	53 (29.4)	38 (21.2)	
Cigarette smoking status (n = 536)					.75				.006				.17
Nonsmoker	231 (43.1)	78 (43.6)	73 (40.6)	80 (45.2)		69 (38.3)	74 (41.3)	88 (49.7)		76 (42.2)	77 (43.0)	78 (44.1)	
Ex-smoker	286 (53.4)	95 (53.1)	102 (56.7)	89 (50.3)		98 (54.4)	102 (57.0)	86 (48.6)		101 (56.1)	97 (54.2)	88 (49.7)	
Current smoker	19 (3.5)	6 (3.4)	5 (2.8)	8 (4.5)		13 (7.2)	3 (1.7)	3(1.7)		3 (1.7)	5 (2.8)	11 (6.2)	
Supplement use (vitamins, minerals and/or fish oil)	105 (19.5)	39 (21.7)	36 (20.0)	30 (16.8)	.49	38 (21.1)	25 (13.9)	42 (23.5)	.058	37 (20.6)	34 (18.9)	34 (19.0)	.91
Number of medications	4.0 (2.0−6.0)	4.0 (2.0−6.0)	3.0 (2.0−5.0)	3.0 (2.0−5.0)	.10	4.0 (2.0−6.0)	3.0 (2.0−5.0)	4.0 (2.0−6.0)	.17	3.0 (2.0−5.0)	3.0 (2.0−6.0)	4.0 (2.0−5.0)	.65
Number of cardiovascular medications	2.0 (1.0−3.0)	2.0 (1.0−3.0)^a,b^	2.0 (1.0−3.0) ^a,c^	2.0 (0.0−2.0) ^b,c^	.020^a^	2.0 (0.0−3.0)	2.0 (0.3−3.0)	2.0 (1.0−3.0)	.81	2.0 (1.0−3.0)	2.0 (1.0−3.0)	2.0 (1.0−3.0)	.51
					.007^b^								
					1.00^c^								
Cardiovascular medications use	416 (77.2)	144 (80.0)	140 (77.8)	132 (73.7)	.36	133 (73.9)	135 (75.0)	148 (82.7)	.10	140 (77.8)	139 (77.2)	137 (76.5)	.96
Anti-coagulant and/or anti-platelet	223 (41.4)	85 (47.2)	73 (40.6)	65 (36.3)	.11	69 (38.3)	69 (38.3)	85 (47.5)	.13	73 (40.6)	72 (40.0)	78 (43.6)	.76
Anti-hypertensives	334 (62.0)	120 (66.7)	108 (60.0)	106 (59.2)	.28	110 (61.1)	111 (61.7)	113 (63.1)	.92	113 (62.8)	111 (61.7)	110 (61.5)	.96
Anti-lipemic	205 (38.0)	79 (43.9)	67 (37.2)	59 (33.0)	.10	67 (37.2)	59 (32.8)	79 (44.1)	.083	74 (41.1)	61 (33.9)	70 (39.1)	.35
Cardiovascular agents	21 (3.9)	9 (5.0)	4 (2.2)	8 (4.5)	.35	8 (4.4)	4 (2.2)	9 (5.0)	.35	7 (3.9)	6 (3.3)	8 (4.5)	.86
Diuretics	32 (5.9)	12 (6.7)	13 (7.2)	7 (3.9)	.36	12 (6.7)	10 (5.6)	10 (5.6)	.88	10 (5.3)	12 (6.7)	10 (5.6)	.88
Anaemia (n = 523) (Haemoglobin < 130 g/L)	76 (14.5)	24 (13.6)	26 (15.2)	26 (14.9)	.90	27 (15.5)	26 (14.7)	23 (13.4)	.85	30 (17.2)	20 (11.5)	26 (14.9)	.31
Haemoglobin (g/L) (n = 523)	144.0 (136.0−152.0)	144.0 (135.0−152.0)	143.0 (136.0−151.0)	145.0 (137.0−154.0)	.26	143.0 (133.8−152.0)	145.0 (136.5−153.0)	145.0 (138.0−153.0)	.50	143.0 (133.0−151.3)^a,b^	146.0 (136.0−152.0) ^a,c^	145.0 (137.0−153.0) ^b,c^	.041^a^
													.44 ^b^
													1.00^c^
Interleukin-6 (pg/mL) (n = 492)	2.5 (1.2−5.0)	2.7 (1.4−5.6)	2.1 (1.1−3.9)	2.7 (1.4−5.4)	.33	2.9 (1.5−5.3)	2.1 (1.1−3.8)	2.7 (1.3−5.6)	.12	2.4 (1.2−4.7)	2.1 (1.3−5.4)	2.9 (1.3−5.0)	.068
MMSE (n = 511)	28.0 (26.0−29.0)	28.0 (27.0−29.0)	28.0 (26.0−30.0)	28.0 (26.8−29.0)	.70	28.0 (26.0−29.0)	28.0 (26.0−29.0)	29.0 (27.0−30.0)	.043†	28.0 (26.0−29.0)	28.0 (27.0−30.0)	28.0 (27.0−29.0)	.53
Cognitively impaired (n = 511)	45 (8.8)	17 (9.9)	14 (8.2)	14 (8.2)	.81	17 (10.8)	20 (11.4)	8 (4.5)	.039	21 (12.7)	11 (6.5)	13 (7.4)	.10
Self-rated health					.63				.15				.47
Very poor/poor/fair	113 (21.0)	42 (23.3)	35 (19.4)	36 (20.1)		46 (25.6)	36 (20.0)	31 (17.3)		35 (19.4)	35 (19.4)	43 (24.0)	
Good/excellent	426 (79.0)	138 (76.7)	145 (80.6)	143 (79.9)		134 (74.4)	144 (80.0)	148 (82.7)		145 (80.6)	145 (80.6)	136 (76.0)	
Number of comorbidities	2.0 (1.0−3.0)	2.0 (1.0−3.0)	2.0 (1.0−3.0)	2.0 (1.0−3.0)	.58	2.0 (1.0−3.0)	2.0 (1.0−3.0)	2.0 (1.0−3.0)	.99	2.0 (1.0−3.0)	2.0 (1.0−3.0)	2.0 (1.0−3.0)	.95
Diabetes Mellitus	105 (19.5)	32 (17.8)	40 (22.2)	33 (18.4)	.52	41 (22.8)	31 (17.2)	33 (18.4)	.38	36 (20.0)	38 (21.1)	31 (17.3)	.65
Chronic Kidney Disease (eGFR <60 mL/min/1.73 m^2^) (n = 525)	171 (32.6)	68 (38.4)	53 (30.6)	50 (28.6)	.12	54 (30.9)	66 (37.3)	51 (29.5)	.25	62 (35.2)	51 (29.3)	58 (33.1)	.49
History of cancer	42 (7.8)	13 (7.2)	15 (8.3)	14 (7.8)	.93	12 (6.7)	13 (7.2)	17 (9.5)	.57	13 (7.2)	13 (7.2)	16 (8.9)	.78
Frailty status (n = 534)					.13				.18				.083
Robust	266 (49.8)	84 (47.5)	83 (46.4)	99 (55.6)		81 (45.0)	95 (53.7)	90 (50.8)		104 (58.4)	83 (46.1)	79 (44.9)	
Pre-frail	246 (46.1)	82 (46.3)	88 (49.2)	76 (42.7)		87 (48.3)	78 (44.1)	81 (45.8)		67 (37.6)	89 (49.4)	90 (51.1)	
Frail	22 (4.1)	11 (6.2)	8 (4.5)	3 (1.7)		12 (6.7)	4 (2.3)	6 (3.4)		7 (3.9)	8 (4.4)	7 (4.0)	
PASE	131.7 (88.5−166.7)	122.4 (78.8−165.1)	135.9 (89.9−167.0)	136.0 (95.7−169.2)	.18	126.1 (81.9−161.5)	140.8 (89.7−171.7)	133.4 (92.0−166.1)	.31	135.9 (81.1−164.3)	129.3 (81.3−167.5)	131.7 (92.0−167.1)	.70

*Notes*: BMI = body mass index; eGFR = estimated glomerular filtration rate; MMSE = Mini-Mental State Examination; PASE = Physical Activity Scale for the Elderly. Data was available for n = 539 for all participant characteristics except where indicated.

1 P values were obtained using the median test and Bonferroni correction for multiple tests to compare all dietary pattern score tertile groups for differences in median values of continuous variables. Differences between groups are denoted by each letter a, b, or c. P values were obtained using the chi-square test to compare all dietary pattern score tertile groups for differences in proportions of participants in categories for categorical variables.

† No differences between groups were observed after Bonferroni correction for multiple tests.

For the ‘vegetables-legumes-seafood’ dietary pattern, the middle tertile had higher intakes than the bottom tertile but lower intakes than the top tertile of other vegetables, dark green vegetables, and red orange vegetables. The middle tertile of ‘vegetables-legumes-seafood’ dietary pattern also had higher intakes of seafood, red meat, other fruits, and poultry, and lower intakes of milk than the bottom tertile. The top tertile of ‘vegetables-legumes-seafood’ dietary pattern had higher intakes of starchy vegetables, legumes, eggs, and nuts and seeds than the bottom tertile.

For the ‘wholegrains-milk-other fruits’ dietary pattern, the middle tertile had higher intakes than the bottom tertile but lower intakes than the top tertile of wholegrains and other fruits. The middle tertile of ‘wholegrains-milk-other fruits’ also had lower intakes than the bottom tertile but higher intakes than the top tertile of refined grains, and higher intakes of nuts and seeds than the bottom tertile. The top tertile of ‘wholegrains-milk-other fruits’ dietary pattern had higher intakes of milk, citrus, melons and berries, discretionary, nuts and seeds, and starchy vegetables than the bottom tertile.

For the ‘discretionary-starchy vegetables-processed meats’ dietary pattern, the top tertile had higher intakes of discretionary, starchy vegetables, processed meats, milk, and refined grains than both bottom and middle tertiles. The top tertile of the ‘discretionary-starchy vegetables-processed meats’ dietary pattern also had higher intakes of red meat, and cheese, and lower intakes of red orange vegetables, seafood, other fruits, and citrus, melons, and berries than the bottom tertile.

### 3.3 Dietary patterns and MACE

**Fig. 3:**
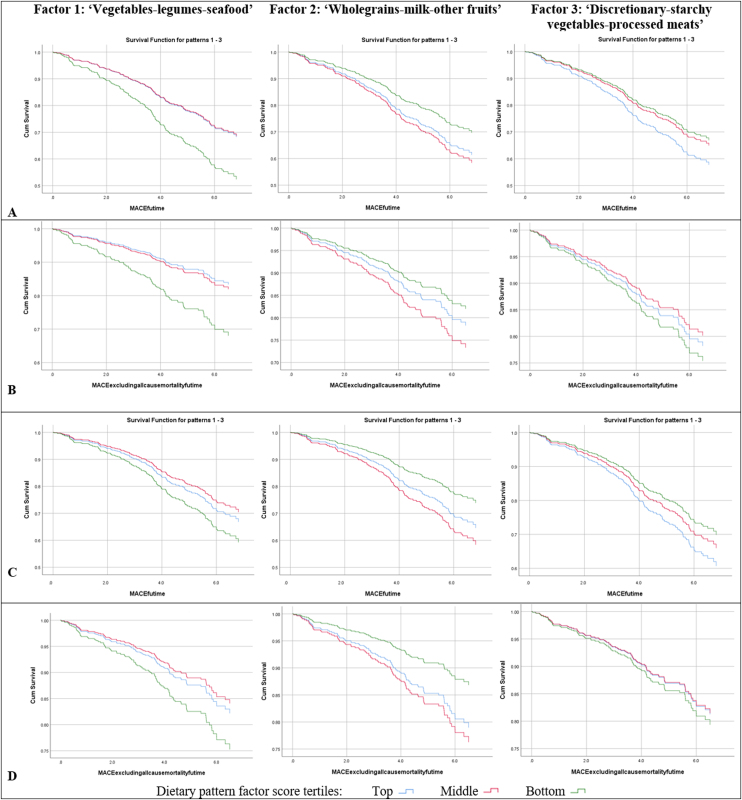
Survival free of MACE: unadjusted analyses (A) Five-point MACE and (B) Four-point MACE excluding all-cause mortality; and fully adjusted analyses (C) Five-point MACE and (D) Four-point MACE excluding all-cause mortality. MACE = major adverse cardiovascular event; Cum = cumulative; Futime = follow-up time.

During the median follow-up of 5.3 (IQR 4.6–6.3) years, the incidences were: five-point MACE 31.2% (n = 168); four-point MACE excluding all-cause mortality 17.8% (n = 96); all-cause mortality 20.1% (n = 111); CCF 11.3% (n = 61); MI 3.7% (n = 20); stroke 3.2% (n = 17); and coronary revascularisation 3.1% (n = 15). The number of events for MACE and individual endpoints of MACE according to tertiles of dietary pattern scores are available in Supplementary Table 3. Univariate analyses of predictors of MACE are shown in Supplementary Table 4. Survival analysis plots on MACE stratified by tertiles of dietary pattern scores are shown in Fig. 3 for unadjusted and fully adjusted analyses. The associations between dietary pattern scores and MACE is presented in Table 2.

**Table 2:** Associations between dietary pattern factor scores and major adverse cardiovascular events (MACE) using Cox regression presented as hazard ratios (95% CI) (n = 539).

Dietary Pattern Factor Scores	Bottom tertile (reference category)	Middle tertile	Top tertile	As continuous variable (per 1 increment)
Factor 1: ‘Vegetables-legumes-seafood’^a^				
**Five-point MACE**				
Model 1	1	0.58 (0.41, 0.84) P = .004	0.59 (0.41, 0.85) P = .005	0.78 (0.66, 0.95) P = .011
Model 2	1	0.65 (0.44, 0.96) P = .029	0.69 (0.47, 1.03) P = .068	0.84 (0.69, 1.03) P = .089
Model 3	1	0.67 (0.45, 0.99) P = .047	0.77 (0.51, 1.16) P = .21	0.88 (0.72, 1.07) P = .19
**Four-point MACE excluding all-cause mortality**				
Model 1	1	0.52 (0.32, 0.84) P = .007	0.47 (0.29, 0.78) P = .003	0.70 (0.54, 0.91) P = .007
Model 2	1	0.54 (0.33, 0.89) P = .016	0.59 (0.34, 1.01) P = .056	0.76 (0.57, 1.01) P = .060
Model 3	1	0.61 (0.36, 1.02) P = .061	0.69 (0.39, 1.21) P = .20	0.82 (0.61, 1.09) P = .17
Factor 2: ‘Wholegrains-milk-other fruits’^b^				
**Five-point MACE**				
Model 1	1	1.49 (1.02, 2.18) P = .040	1.35 (0.92, 1.99) P = .13	1.06 (0.91, 1.24) P = .45
Model 2	1	1.54 (1.02, 2.33) P = .041	1.38 (0.88, 2.18) P = .16	1.04 (0.87, 1.24) P = .68
Model 3	1	1.78 (1.17, 2.70) P = .007	1.44 (0.91, 2.30) P = .12	1.05 (0.87, 1.25) P = .63
**Four-point MACE excluding all-cause mortality**				
Model 1	1	1.56 (0.95, 2.57) P = .078	1.23 (0.73, 2.08) P = .43	1.04 (0.85, 1.27) P = .72
Model 2	1	1.72 (1.01, 2.93) P = .044	1.73 (0.94, 3.18) P = .078	1.13 (0.88, 1.46) P = .35
Model 3	1	1.92 (1.12, 3.30) P = .018	1.67 (0.90, 3.13) P = .11	1.14 (0.87, 1.48) P = .34
Factor 3: ‘Discretionary-starchy vegetables-processed meats’^c^				
**Five-point MACE**				
Model 1	1	1.07 (0.73, 1.58) P = .72	1.37 (0.95, 1.97) P = .096	1.29 (1.10, 1.51) P = .002
Model 2	1	1.12 (0.74, 1.68) P = .60	1.44 (0.93, 2.21) P = .10	1.36 (1.12, 1.64) P = .002
Model 3	1	1.16 (0.76, 1.77) P = .49	1.39 (0.89, 2.18) P = .14	1.33 (1.09, 1.62) P = .004
**Four-point MACE excluding all-cause mortality**				
Model 1	1	0.78 (0.48, 1.28) P = .33	0.87 (0.54, 1.40) P = .57	1.14 (0.93, 1.40) P = .21
Model 2	1	0.86 (0.51, 1.45) P = .58	0.96 (0.55, 1.68) P = .90	1.26 (0.98, 1.62) P = .074
Model 3	1	0.89 (0.52, 1.51) P = .66	0.90 (0.50, 1.60) P = .71	1.24 (0.95, 1.60) P = .11

*Notes*: Model 1 unadjusted (n = 539 for total, 168 five-point MACE, and 96 four-point MACE excluding all-cause mortality); Model 2 adjusted by sociodemographic and lifestyle factors (age (continuous), BMI (continuous), country of birth (Australia v. Greece/Italy v. other), source of income (Age Pension only v. other), marital status (married/de facto v. not married/divorced/separated/widowed/never married/other), smoking status (nonsmoker v. ex-smoker v. current smoker), energy intake (continuous), supplement use including vitamins, minerals, and/or fish oil (yes v. no)) (n = 526 for total, 160 five-point MACE, and 95 four-point MACE excluding all-cause mortality); Model 3 adjusted by Model 2 plus health (haemoglobin (continuous), number of cardiovascular medications (continuous), frailty status (robust v. pre-frail v. frail), history of cancer (yes v. no) and CKD (yes v. no)) (n = 510 for total, 160 five-point MACE, and 92 four-point MACE excluding all-cause mortality).

a Bottom tertile ≤ −0.48, n = 180; middle tertile −0.47 to 0.21, n = 180; top tertile ≥ 0.22, n = 179.

b Bottom tertile ≤ −0.41, n = 180; middle tertile −0.40 to 0.40, n = 180; top tertile ≥ 0.41, n = 179.

c Bottom tertile ≤ −0.38, n = 180; middle tertile −0.37 to 0.40, n = 180; top tertile ≥ 0.41, n = 179.

***‘Vegetables-legumes-seafood’***

Compared with the bottom tertile, both the middle and top tertiles of the ‘vegetables-legumes-seafood’ dietary pattern score were associated with reduced risks of five-point MACE (HR 0.58 [95% CI: 0.41, 0.84, P = .004] and HR 0.59 [95% CI: 0.41, 0.85, P = .005]) and four-point MACE excluding all-cause mortality (HR 0.52 [95% CI: 0.32, 0.84, P = .007] and HR 0.47 [95% CI: 0.29, 0.78, P = .003]) in unadjusted analyses. However, in fully adjusted analyses only the middle tertile remained associated with reduced risk of five-point MACE (HR 0.67 [95% CI: 0.45, 0.99, P = .047]). A higher ‘vegetables-legumes-seafood’ dietary pattern score (per 1 increment) was associated with reduced risks of five-point MACE and four-point MACE excluding all-cause mortality in unadjusted analyses (HR 0.78 [95% CI: 0.66, 0.95, P = .011] and HR 0.70 [95% CI: 0.54, 0.91, P = .007]), but were not associated in fully adjusted analyses.

***‘Wholegrains-milk-other fruits’***

Compared with the bottom tertile, the middle tertile of the ‘wholegrains-milk-other fruits’ dietary pattern score was associated with increased risk of five-point MACE in unadjusted analyses (HR 1.49 [95% CI: 1.02, 2.18, P = .040]). In fully adjusted analyses, the middle tertile of the ‘wholegrains-milk-other fruits’ dietary pattern score was associated with increased risk of five-point MACE and four-point MACE excluding all-cause mortality (HR 1.78 [95% CI: 1.17, 2.70, P = .007] and HR 1.92 [95% CI: 1.12, 3.30, P = .018], respectively). A higher ‘wholegrains-milk-other fruits’ dietary pattern score (per 1 increment) was not associated with five-point MACE and four-point MACE excluding all-cause mortality in unadjusted and fully adjusted analyses.

***‘Discretionary-starchy vegetables-processed meats’***

A higher ‘discretionary-starchy vegetables-processed meats’ dietary pattern score (per 1 increment) was associated with increased risks of five-point MACE in unadjusted analyses (HR 1.29 [95% CI: 1.10, 1.51, P = .002]), which remained associated in fully adjusted analyses (HR 1.33 [95% CI: 1.09, 1.62, P = .004]). The ‘discretionary-starchy vegetables-processed meats’ dietary pattern score as categorical variables was not associated with five-point MACE, and as both categorical and continuous variables was not associated with four-point MACE excluding all-cause mortality in unadjusted and fully adjusted analyses.

### 3.4 Subanalyses of dietary patterns and individual endpoints of MACE

Survival analysis plots on individual endpoints of MACE stratified by tertiles of dietary pattern scores are shown in Supplementary Fig. 2 for unadjusted adjusted analyses and Supplementary Fig. 3 for fully adjusted analyses. The associations between dietary pattern scores and individual endpoints of MACE is presented in Supplementary Table 5. In fully adjusted analyses, the middle tertile of the ‘vegetables-legumes-seafood’ dietary pattern score remained associated with reduced risk of CCF (HR 0.31 [95% CI: 0.15, 0.65, P = .002]), whilst the middle tertile of the ‘wholegrains-milk-other fruits’ dietary pattern score remained associated with increased risk of CCF (HR 2.33 [95% CI: 1.17, 4.65, P = .016]). The top tertile of and a higher ‘discretionary-starchy vegetables-processed meats’ dietary pattern score (per 1 increment) remained associated with increased risks of all-cause mortality in fully adjusted analyses (HR 2.26 [95% CI: 1.27, 4.00, P = .005]) and HR 1.63 [95% CI: 1.26, 2.12, P < .001] respectively).

### 3.5 Subgroup analyses by age of dietary patterns, MACE, and individual endpoints of MACE

The subgroup analyses by age of associations between dietary pattern scores, MACE, and individual endpoints of MACE is presented in Supplementary Table 6. In adjusted analyses, the middle tertile of the ‘vegetables-legumes-seafood’ dietary pattern score remained associated with reduced five-point MACE, four-point MACE excluding all-cause mortality, and CCF in older men aged >85 years but not in older men aged 75–84 years (HR 0.17 [95% CI: 0.07, 0.47, P = .001]), HR 0.09 [95% CI: 0.02, 0.42, P = .002] and HR 0.09 [95% CI: 0.01, 0.68, P = .019] respectively). A higher ‘vegetables-legumes-seafood’ dietary pattern score remained associated with reduced CCF in older men aged >85 years but not older men aged 75–84 years in adjusted analyses (HR 0.30 [95% CI: 0.11, 0.80, P = .016]). The middle tertile of the ‘wholegrains-milk-other fruits’ dietary pattern score was associated with increased five point-MACE, four-point MACE excluding all-cause mortality, all-cause mortality, and CCF in older men aged 75–84 years but not in older men >85 years in adjusted analyses (HR 2.37 [95% CI: 1.41, 3.99, P = .001]), HR 3.20 [95% CI: 1.60, 6.43, P = .001], HR 2.20 [95% CI: 1.13, 4.27, P = .020] and HR 5.54 [95% CI: 1.93, 15.86, P = .001] respectively). The top tertile of the ‘wholegrains-milk-other fruits’ dietary pattern score was also associated with increased four-point MACE excluding all-cause mortality, and CCF in older men aged 75–84 years but not in older men >85 years in adjusted analyses (HR 2.50 [95% CI: 1.13, 5.53, P = .024] and HR 3.79 [95% CI: 1.20, 11.96, P = .023] respectively). A higher ‘discretionary-starchy vegetables-processed meats’ dietary pattern score remained associated with increased five-point MACE, all-cause mortality, and coronary revascularisation in older men aged 75–84 years but not in older men aged >85 years in adjusted analyses (HR 1.36 [95% CI: 1.06, 1.75, P = .017], HR 1.59 [95% CI: 1.15, 2.21, P = .006] and HR 2.42 [95% CI: 1.14, 5.11, P = .021] respectively).

## 4 Discussion

To the authors’ knowledge, this is the first study to examine the longitudinal associations between empirically derived dietary patterns with MACE in older men aged 75 years and over. We found that the middle tertile of the ‘vegetables-legumes-seafood’ dietary pattern score was associated with reduced risks of five-point MACE and CCF in fully adjusted analyses, whilst the middle tertile of the ‘wholegrains-milk-other fruits’ dietary pattern score was associated with increased risks of five-point MACE, four-point MACE excluding all-cause mortality, and CCF. A higher ‘discretionary-starchy vegetables-processed meats’ dietary pattern score was associated with increased risks of five-point MACE and all-cause mortality, and the top tertile was also associated with increased risk of all-cause mortality. There were differences in the associations between the empirically derived dietary patterns with MACE and individual endpoints of MACE by age subgroups of older men aged 75–84 years and >85 years.

Similar to the middle tertile of the ‘vegetables-legumes-seafood’ dietary pattern, other dietary patterns consisting of higher intakes of vegetables, seafood, and other fruits have been associated with protective health benefits. In randomised controlled trials, those on the Mediterranean diet had reduced risk of developing MACE over 4.8 years in adults aged 55–80 years [32], and improved cardiometabolic health parameters over 3–6 months in older adults aged 65 years and over [4,33,34]. A systematic review and meta-analysis involving 17 prospective cohort studies found that in comparison to the lowest category, the highest category of empirically derived prudent or healthy dietary patterns with high factor loadings of vegetables, fruits, legumes, wholegrains, fish and poultry, were longitudinally associated with reduced risks of CVD and CHD (coronary heart disease) in adults aged 18 years and over with a follow-up range of 2–18 years [13]. These findings have been similarly reflected in previous research through diet quality indices: the Mediterranean diet has been longitudinally associated with reduced risks of developing MACE over 4.7 years in older men aged 75 years and over in the CHAMP cohort [11], reduced risks of all-cause and CVD mortality in men aged 50 years and over with a follow-up of 9.8 years [35] and in older adults aged 70–90 years with a follow-up of 10 years [36]; the Dietary Approaches to Stop Hypertension (DASH) diet has been longitudinally associated with reduced risk of all-cause mortality in men aged 50 years and over with a follow-up of 9.8 years [35], reduced risks of CVD, CHD, and MACE in adults aged 18 years and over with a follow-up range of 7–24 years [37], and reduced risk of developing CCF over 9 years in men aged 45–79 years [38]; healthful plant-based diets have been longitudinally associated with reduced risk of CVD in adults aged 18 years and over with a follow-up range of up to 32 years [39]; the revised Dietary Guideline Index has been cross-sectionally associated with better cardiometabolic parameters in older men aged 75 years and over in the CHAMP cohort [10]; the Healthy Eating Index–2010 and Alternative Healthy Eating Index–2010 have been associated with reduced risks of all-cause mortality and CVD mortality in older men aged 50 years and over with a follow-up of 9.8 years [35].

Importantly, the results of this study showed that empirically derived dietary patterns can provide insight on how the interactions between foods consumed habitually in the population can influence associations. Although the middle tertile of the ‘vegetables-legumes-seafood’ dietary pattern also had higher intakes of red meat than the bottom tertile, a food group associated with increased risks of CVD mortality, the high consumption of cardioprotective foods such as vegetables may have attenuated the association [40]. Thus the middle tertile of the ‘vegetables-legumes-seafood’ dietary pattern was associated with reduced risks of five-point MACE and CCF. The top tertile of the ‘vegetables-legumes-seafood’ dietary pattern score also had higher intakes of starchy vegetables and eggs, and was not associated with five-point MACE, four-point MACE excluding all-cause mortality, and individual endpoints of MACE. A meta-analysis found a lack of association between starchy vegetables or potatoes with all-cause and CVD mortality in adults aged 18 years and over [41], which could be due to the higher glycaemic index or glycaemic load that has been longitudinally associated with increased risks of type 2 diabetes mellitus [42], a risk factor for CVD. Furthermore, there has been conflicting associations between egg consumption with MACE [43,44], CHD [45], and CCF [46].

The middle tertile of the ‘wholegrains-milk-other fruits’ dietary pattern score was associated with increased risks of five-point MACE, four-point MACE excluding all-cause mortality, and CCF, whilst the top tertile of the ‘wholegrains-milk-other fruits’ dietary pattern score was not associated with five-point MACE, four-point MACE excluding all-cause mortality, and individual endpoints of MACE which were unexpected findings. A systematic analysis of the Global Burden of Disease study found that low intakes of wholegrains, fruits, nuts and seeds were leading contributors of diet-attributable CVD mortality in adults aged 18 years and over [47]. Furthermore, wholegrains, fruits, nuts and seeds are common components of the previously mentioned Mediterranean, DASH, and healthy plant-based dietary patterns associated with reduced risks of MACE [11,32,37], CVD [37,39], CCF [38], and improved cardiometabolic health parameters [33,34]. The lack of association for the top tertile of the ‘wholegrains-milk-other fruits’ dietary pattern could be explained by the higher intakes of starchy vegetables [41], and the higher intakes of discretionary such as sugar-sweetened beverages that have been associated with increased risks of all-cause and CVD mortality in men aged 40–75 years with a follow-up of 28 years [48]. The increased risk of five-point MACE, four-point MACE excluding all-cause mortality, and CCF from consuming the middle tertile of the ‘wholegrains-milk-other fruits’ dietary pattern score requires further investigation. Future research, could increase the number of food groupings used to derive the empirically derived dietary pattern which may provide more detail on particular food items that could have influenced this association.

A higher ‘discretionary-starchy vegetables-processed meats’ dietary pattern score was associated with increased risks of five-point MACE and all-cause mortality, and the top tertile was also associated with increased risk of all-cause mortality. This is consistent with previous research on empirically derived dietary patterns in other populations and age groups: a dietary pattern characterised by sweets, red meat, margarine, salty nuts, hard cheese, and alcohol was longitudinally associated with increased risk of CVD in adults aged 18 years and over with a follow-up of 5 years [49], the ‘Southern pattern’ characterised by added fats, fried food, eggs, organ meats, processed meats, and sugar-sweetened beverages, was longitudinally associated with increased risk of CHD in adults aged 45 years and over with a follow-up of 5.8 years [50], the ‘Western pattern’ with higher intakes of red meat, processed meat, refined grains, sweets and dessert, French fries, and high-fat dairy products, were longitudinally associated with increased risk of developing CHD over 8 years in men aged 40–75 years [51], and the ‘high fat/low fibre’ dietary pattern high in red meat, meat products, white bread, fried potato, and eggs was longitudinally associated with increased risk of all-cause mortality in men aged 60–79 years with a follow-up of 11 years [52].

There are several study limitations. As our study is observational, causation cannot be determined from longitudinal analyses on dietary pattern scores with MACE, and residual confounding cannot be ruled out. Dietary exposure and other measures were only from a single timepoint that may have changed over the follow-up period, possibly resulting in misclassification of exposure factors to some extent but which would be expected to nullify rather than create positive findings. Food groupings were obtained using FoodWorks which is based on the Australian Guide to Healthy Eating, and we used an adapted definition for a serve of discretionary which included sugar, solid fats, and alcohol. In factor analysis, there is potential subjectivity in the number of factors retained as they are based on empirical guidelines rather than exact quantitative solutions, in the labelling of the dietary patterns and the groupings of food items [5,[50], [51], [52]]. However, the food groups used in the present study were based on those converted from FoodWorks. The three dietary patterns derived explained 23.9% of the total variance suggests there is heterogeneity amongst dietary intake in this cohort of older men and the potential existence of other dietary patterns. However, it is similar to the total variance of prior studies [51,52], and the ‘percentage of variance explained’ should be interpreted with caution as it is a function of the number of food group items included in the factor analysis: as food is more broadly classified with a lower number of food groups, the greater the total variance explained by resultant dietary patterns [51]. All-cause mortality was included in the composite index of five-point MACE as data on cause-specific mortality was not available. However, we also presented analyses with four-point MACE excluding all-cause mortality [53]. Data on family history of CVD, which could influence CVD risk from attributions of shared genetic and lifestyle factors, was not collected [54]. Thus genetic predisposition was not accounted for. However, lifestyle factors were collected and adjusted for in analyses. Furthermore, previous research has shown that family history of CVD as a predictor of CVD risk reduced with increasing age [54] and family history of MI was not a significant predictor of incident MACE in older adults [55]. Subgroup analyses by age between empirically derived dietary patterns and individual endpoints of MACE were limited by the reduced number of events per subgroup. Our study was limited to community-dwelling men and the dietary patterns were derived from this study population, therefore our results may not apply to older women or institutionalised populations.

The strength of our study is that we explored the longitudinal associations between dietary pattern scores with MACE over time. Examining empirically derived dietary pattern scores as exposure rather than diet quality indices, allows for the exploration of dietary patterns independent of current knowledge [7]. We used a validated dietitian-administered diet history questionnaire, which has been indicated for older adults due to the non-reliance on short-term memory and low respondent burden [16]. We considered multiple confounders for inclusion in the model, and adjustments included the number of cardiovascular medications (anti-coagulants and antiplatelets (including aspirin), anti-hypertensive agents (beta-adrenergic blockers, hypotensive agents, and vasodilating agents), cardiovascular agents (cardiac agents and cardiac glycosides), antilipemic agents, and diuretics) that could impact on the development of MACE and dietary intake, and energy intake that would also account for under or over-reporting. A further strength of CHAMP is that it includes a large and representative group of older Australian men [8].

## 5 Conclusion

The middle tertile of the ‘all-vegetables-legumes-seafood’ dietary pattern was associated with reduced risks, whilst the middle tertile of the ‘wholegrains-milk-other fruits’, top tertile and a higher ‘discretionary-starchy vegetables-processed meats’ dietary pattern were associated with increased risks of developing MACE over 5 years in older men. Older men may benefit from consuming a ‘vegetables-legumes-seafood’ dietary pattern rather than ‘discretionary-starchy vegetables-processed meats’ and ‘wholegrains-milk-other fruits’ dietary patterns for the prevention of MACE.

## Conflict of interest

The authors declare no conflicts of interest.

## Funding

The CHAMP study is funded by the 10.13039/501100000925National Health and Medical Research Council (project grant no. 301916), Ageing and Alzheimers Research Institute, Ageing and Alzheimers Research Foundation, and the 10.13039/501100007811Sydney Medical School Foundation. This research was supported by the Australian Research Council Centre of Excellence in Population Ageing Research (project number CE170100005). RL was supported by an Australian Government Research Training Program Scholarship and an Australian Research Council Centre of Excellence in Population Aging Research Scholarship. RR was supported by a Charles Perkins Centre Early Career Fellowship from Jennie Mackenzie. The funders were not involved in the conception, design, performance, or approval of this work.

## Author contributions

R.L., R.R., V.N., and V.H. conceptualised the analyses presented in this manuscript. R.L., R.R., and V.H. designed the analyses. R.L. performed the data analyses. R.L., R.R., and V.H. interpreted the data. R.L. wrote the manuscript. V.N., F.M.B., L.M.W., D.J.H., D.G.L.C., M.J.S., and V.H. were principal investigators in the original and succeeding waves of the Concord Health and Ageing in Men Project, each contributing to design of the Project, data collection, and analysis. All authors played a critical role in reviewing, editing and approved the final version of the manuscript. R.L. and V.H. had full access to all of the data in the study and take responsibility for the integrity of the data and the accuracy of the data analysis.

## Ethical standards

The CHAMP study was conducted according to the guidelines laid down in the Declaration of Helsinki and all procedures involving human subjects were approved by the Concord Hospital Human Research Ethics Committee (HREC/14/CRGH/17). Written informed consent was obtained from all participants.
