# Supplementary material for: Empirically derived dietary patterns are associated with major adverse cardiovascular events, all-cause mortality, and congestive cardiac failure in older men: The Concord Health and Ageing in Men Project
Source: J Nutr Health Aging. 2024 Jan 1;28(2):100020. doi: 10.1016/j.jnha.2023.100020 (PMC12877253; doi:10.1016/j.jnha.2023.100020)
Supplement: Supplementary file 1 [file mmc1.pdf]

I. **Supplementary Table 1.** Factor loadings with varimax rotation to determine the association between 23 food groups and factors representing dietary patterns among older Australian men (n = 539)

II. **Supplementary Table 2.** Dietary intake (median and interquartile range) according to dietary pattern score tertiles (n = 539)

III. **Supplementary Table 3.** Number (%) of events of major adverse cardiovascular events (MACE) and individual endpoints of MACE stratified by tertiles of dietary pattern scores (n = 539)

IV. **Supplementary Table 4.** Predictors of five-point MACE in univariate analysis unadjusted using Cox regression presented as hazard ratios (95% CI) (n=539)

V. **Supplementary Table 5.** Associations between dietary pattern factor scores and individual endpoints of MACE using Cox regression presented as hazard ratios (95% CI) (n = 539)

VI. **Supplementary Table 6.** Subgroup analyses of associations between dietary pattern factor scores, MACE and individual endpoints of MACE using Cox regression presented as hazard ratios (95% CI) (n = 539)

VII. **Supplementary Figure 1.** Scree plot for the factor analysis

VIII. **Supplementary Figure 2.** Probability of MACE-free survival curves based on dietary pattern score tertiles in unadjusted analyses: (A) all-cause mortality; (B) CCF; (C) coronary revascularisation; (D) MI; (E) stroke. MACE = major adverse cardiovascular event; CCF = congestive cardiac failure; revas = coronary revascularisation; MI = myocardial infarction; Cum = cumulative; Futime = follow-up time.

IX. **Supplementary Figure 3.** Probability of MACE-free survival curves based on dietary pattern score tertiles in fully adjusted analyses: (A) all-cause mortality; (B) CCF; (C) coronary revascularisation; (D) MI; (E) stroke. MACE = major adverse cardiovascular event; CCF = congestive cardiac failure; revas = coronary revascularisation; MI = myocardial infarction; Cum = cumulative; Futime = follow-up time.

Supplementary Table 1. Factor loadings with varimax rotation to determine the association between 23 food groups and factors representing dietary patterns among older Australian men (n = 539)

**Foods**	**Factor 1: 'Vegetables-legumes-seafood'**	**Factor 2: 'Wholegrains-milk-other fruits'**	**Factor 3: 'Discretionary-starchy vegetables-processed meats'**
Other vegetables	**0.76**	0.08	0.06
Dark green vegetables	**0.65**	-0.17	-0.17
Red orange vegetables	**0.54**	0.21	-0.11
Legumes	**0.35**	-0.20	-0.09
Seafood	**0.32**	0.06	-0.19
Red meats	0.30	-0.10	0.23
Wholegrains	0.02	**0.70**	0.03
Refined grains	0.16	**-0.60**	0.20
Soy products	0.29	**-0.48**	-0.11
Milk	-0.06	**0.39**	**0.38**
Other fruits	0.24	**0.39**	-0.12
Yoghurt	0.16	0.28	-0.02
Nuts and seeds	0.09	0.16	0.00
Fruit juice	-0.03	0.09	0.04
Discretionary	-0.08	0.20	**0.67**
Milk alternatives	0.03	0.15	**-0.47**
Starchy vegetables	**0.39**	0.10	**0.46**
Processed meats	-0.08	0.08	**0.42**

Citrus, melons and berries	0.06	0.25	**-0.41**
Eggs	0.22	0.04	0.24
Cheese	-0.02	0.01	0.14
Organ meats	-0.03	0.01	0.14
Poultry	0.12	0.09	-0.13
% variance explained	9.13	7.73	7.07
Eigen value	2.10	1.78	1.63

Factor loadings with absolute value >0.30 and <-0.30 are shown in bold to indicate major contributing food groups and non-contributing food groups for each dietary pattern respectively.

Supplementary Table 2. Dietary intake (median and interquartile range) according to dietary pattern score tertiles (n = 539)

		**Factor 1: 'Vegetables-legumes-seafood'**				**Factor 2: 'Wholegrains-milk-other fruits'**				**Factor 3: 'Discretionary-starchy vegetables-processed meats'**			
**Variables**	**All n = 539**	**Bottom n = 180**	**Middle n = 180**	**Top n = 179**	**P value[1]**	**Bottom n = 180**	**Middle n = 180**	**Top n = 179**	**P value[1]**	**Bottom n = 180**	**Middle n = 180**	**Top n = 179**	**P value[1]**
Energy intake (kJ)	8862.9 (7312.2-10549.7)	8185.8 (6871.1-9636.9)[a,b]	8470.7 (7274.4-9829.3)[a,c]	9949.9 (8551.1-11526.6)[b,c]	1.00[a] <.001[b,c]	8056.9 (6733.9-9956.5)[a,b]	8263.2 (7218.2-9813.8)[a,c]	9789.6 (8750.7-11508.6)[b,c]	.89[a] <.001[b,c]	7655.9 (6589.3-9073.3)[a,b]	8673.1 (7252.9-10002.0)[a,c]	102778.6 (8993.2-11700.6)[b,c]	.002[a] <.001[b,c]
Nutrients													
Protein (g/kg body weight)	1.3 (1.0-1.6)	1.1 (0.9-1.4)[a,b]	1.2 (1.0-1.5)[a,c]	1.5 (1.3-1.8)[b,c]	.005[a] <.001[b,c]	1.2 (0.9-1.5)[a,b]	1.2 1.0-1.5)[a,c]	1.5 (1.2-1.8)[b,c]	1.00[a] <.001[b,c]	1.1 (0.9-1.4)[a,b]	1.3 (1.0-1.)[a,c]	1.4 (1.2-1.7)[b,c]	.42[a] <.001[b] .001[c]
Protein (g)	100.4 (84.7-119.0)	89.4 (73.9-102.3)[a,b]	96.9 (83.9-115.0)[a,c]	114.8 (101.8-137.4)[b,c]	.018[a] <.001[b,c]	93.5 (75.0-112.7)[a,b]	97.1 (82.4-113.0)[a,c]	111.2 (96.1-129.7)[b,c]	0.62[a] <.001[b,c]	88.3 (73.3-103.7)[a,b]	98.7 (84.9-117.4)[a,c]	112.8 (99.7-128.8)[b,c]	.001[a] <.001[b,c]

Protein (%E)	19.2 (17.0-22.2)	18.5 (16.2-21.2)[a,b]	19.1 (16.9-26.1)[a,c]	20.3 (17.7-23.0)[b,c]	.88[a] .001[b] .31[c]	19.6 (17.0-23.0)	19.1 (17.0-21.7)	18.8 (17.0-22.0)	.40	20.3 (17.2-22.9)[a,b]	19.2 (16.9-22.5)[a,c]	18.7 (16.8-21.0)[b,c]	.061[a] .008[b] .25[c]
Carbohydrate (g)	201.6 (163.9-242.1)	192.8 (151.3-229.9)[a,b]	192.9 (158.2-231.1)[a,c]	226.7 (183.3-267.1)[b,c]	1.00[a] <.001[b,c]	182.3 (140.3-221.7)[a,b]	183.6 (155.5-221.4)[a,c]	237.6 (199.5-276.9)[b,c]	1.00[a] <.001[b,c]	179.9 (144.4-211.5)[a,b]	194.7 (167.0-230.7)[a,c]	237.0 (195.0-276.2)[b,c]	.42[a] <.001[b,c]
Carbohydrate (%E)	37.4 (31.8-42.6)	38.1 (31.4-43.3)	36.5 (31.6-42.5)	37.2 (32.2-41.6)	.34	35.8 (29.9-42.1)[a,b]	35.5 (31.0-41.1)[a,c]	40.0 (35.0-43.6)[b,c]	1.00[a] .003[b] <.001[c]	38.6 (31.1-44.2)	36.2 (31.1-41.6)	37.3 (32.9-41.6)	.17
Added sugar (g)	31.3 (15.0-51.0)	36.5 (17.6-54.3)	29.3 (16.1-48.7)	28.2 (12.8-49.3)	.035†	26.9 (12.9-44.8)[a,b]	28.4 (15.1-49.3)[a,c]	37.5 (21.7-58.8)[b,c]	1.00[a] .011[b] .039[c]	18.0 (9.6-30.8)[a,b]	31.1 (16.3-46.1)[a,c]	50.3 (32.2-69.7)[b,c]	<.001[a,b,c]
Added sugar (%E)	5.9 (3.1-9.3)	7.5 (3.9-10.4)[a,b]	5.7 (3.1-9.0)[a,c]	4.7 (2.4-8.4)[b,c]	.009[a] <.001[b] .25[c]	5.3 (2.9-8.9)	5.7 (3.1-9.4)	6.6 (3.6-9.4)	.14	3.8 (2.1-6.5)[a,b]	5.8 (3.2-8.8)[a,c]	8.3 (4.9-11.2)[b,c]	<.001[a,b] .006[c]
Total fat (g)	84.5 (64.2-108.2)	79.0 (62.6-97.6)[a,b]	82.6 (62.4-105.7)[a,c]	93.4 (68.6-114.5)[b,c]	1.00[a] .021[b] .070[c]	78.3 (58.2-103.0)[a,b]	80.7 (65.5-105.6)[a,c]	93.5 (67.6-116.9)[b,c]	1.00[a] .001[b] .006[c]	69.4 (51.7-93.2)[a,b]	83.3 (64.4-109.7)[a,c]	97.4 (77.8-121.5)[b,c]	.009[a] <.001[b] .003[c]

Total fat (%E)	35.2 (30.0-40.9)	36.1 (30.2-42.5)	34.9 (30.4-41.3)	34.4 (29.6-39.1)	.040[†]	35.2 (29.5-41.8)	36.1 (31.6-41.8)	34.5 (29.5-39.1)	.31	33.4 (28.3-41.1)[a,b]	36.1 (31.2-41.4)[a,c]	35.6 (31.5-40.1)[b,c]	.018[a] .039[b] 1.00[c]
Saturated fat (g)	28.5 (21.6-37.0)	30.0 (22.2-36.3)	27.6 (20.6-35.8)	28.7 (22.2-38.7)	.27	25.3 (19.0-33.2)[a,b]	28.6 (22.3-35.1)[a,c]	32.3 (24.2-40.9)[b,c]	.005[a] <.001[b] .12[c]	21.5 (17.5-27.5)[a,b]	29.3 (22.9-36.6)[a,c]	36.3 (28.5-43.4)[b,c]	<.001[a,b.c]
Saturated fat (%E)	11.9 (9.9-14.5)	13.4 (11.1-15.4)[a,b]	11.8 (10.0-14.1)[a,c]	11.0 (9.0-12.9)[b,c]	.002[a] <.001[b] .091[c]	11.4 (9.3-14.1)[a,b]	12.4 (10.8-14.8)[a,c]	11.9 (9.9-14.5)[b,c]	.005[a] .31[b] .80[c]	10.6 (8.6-12.4)[a,b]	12.2 (10.3-15.2)[a,c]	13.3 (11.2-15.4)[b,c]	<.001[a,b] .021[c]
Monounsaturated fat (g)	34.4 (25.1-47.2)	31.1 (23.0-41.8)[a,b]	33.6 (24.8-45.8)[a,c]	39.1 (28.2-52.3)[b,c]	1.00[a] .003[b] .12[c]	32.4 (23.1-47.2)	33.9 (26.2-44.6)	37.8 (25.6-49.1)	.11	30.8 (20.9-41.4)	34.3 (24.2-45.2)	38.7 (28.9-50.4)	.28[a] <.001[b] .68[c]
Polyunsaturated fat (g)	12.5 (8.5-16.8)	10.7 (7.5-15.5)[a,b]	11.7 (8.4-15.9)[a,c]	13.7 (10.3-19.0)[b,c]	1.00[a] <.001[b] .021[c]	11.0 (7.3-15.3)[a,b]	11.7 (8.3-16.5)[a,c]	13.7 (10.1-18.9)[b,c]	1.00[a] <.001[b] .12[c]	11.5 (7.6-16.1)[a,b]	11.9 (8.7-17.1)[a,c]	13.3 (9.6-17.3)[b,c]	1.00[a] .011[b] .039[c]
Linoleic acid (g)	9.9 (6.8-13.9)	8.4 (5.5-12.7)[a,b]	9.4 (6.6-12.9)[a,c]	11.3 (7.9-15.2)[b,c]	1.00[a] .003[b] .011[c]	9.0 (5.6-12.7)[a,b]	9.2 (6.7-13.5)[a,c]	11.1 (7.9-15.2)[b,c]	1.00[a] <.001[b] .070[c]	9.1 (5.9-13.1)[a,b]	9.4 (6.9-14.4)[a,c]	10.7 (7.3-14.9)[b,c]	1.00[a] .039[b] .070[c]
Linoleic acid (%E)	4.1 (3.1-5.5)	4.0 (3.0-5.3)	4.0 (3.1-5.6)	4.4 (3.3-5.6)	.37	3.8 (2.9-5.5)	4.1 (3.3-5.5)	4.3 (3.2-5.3)	.076	4.2 (3.3-5.7)	4.1 (3.1-5.3)	3.9 (3.0-5.1)	.42

Alpha-linolenic acid (g)	1.2 (0.9-1.9)	1.1 (0.8-1.9)[a,b]	1.2 (0.9-1.8)[a,c]	1.4 (1.0-2.1)[b,c]	1.00[a] .12[b] .001[c]	1.1 (0.7-1.7)[a,b]	1.2 (0.8-2.0)[a,c]	1.5 (1.1-2.1)[b,c]	1.00[a] <.001[b] .021[c]	1.1 (0.7-1.7)	1.2 (0.9-1.9)	1.5 (1.0-2.2)	.061[a] <.001[b] .011[c]
Alpha-linolenic acid (%E)	0.5 (0.4-0.8)	0.5 (0.4-0.8)	0.5 (0.4-0.8)	0.5 (0.4-0.8)	.75	0.5 (0.4-0.7)	0.5 (0.4-0.8)	0.6 (0.4-0.8)	.095	0.5 (0.4-0.8)	0.5 (0.4-0.8)	0.5 (0.4-0.8)	.68
Long chain omega-3 (mg)	486.0 (295.8-728.2)	356.9 (211.1-561.5)[a,b]	539.9 (334.5-731.3)[a,c]	600.2 (371.6-919.6)[b,c]	<.001[a,b] .46[c]	498.8 (291.9-725.6)	430.0 (270.6-680.6)	541.3 (329.2-794.7)	.094	524.7 (279.4-777.2)	473.5 (284.1-691.8)	467.8 (318.8-728.2)	.41
Cholesterol (mg)	301.3 (234.1-379.5)	258.0 (211.5-327.8)[a,b]	288.9 (231.9-368.0)[a,c]	353.6 (276.7-436.0)[b,c]	.034[a] <.001[b] .003[c]	274.4 (229.9-373.1)	296.5 (228.6-373.0)	319.3 (248.7-400.4)	.044[†]	242.5 (188.8-307.9)[a,b]	301.4 (239.8-362.7)[a,c]	365.7 (284.6-460.2)[b,c]	<.001[a,b,c]
Dietary fibre (g)	26.1 (21.1-32.3)	21.3 (16.4-26.1)[a,b]	25.8 (21.8-30.9)[a,c]	32.6 (27.1-40.0)[b,c]	<.001[a,b,c]	21.1 (16.8-27.2)[a,b]	25.2 (21.3-29.2)[a,c]	32.0 (26.1-37.9)[b,c]	<.001[a,b,c]	26.0 (20.9-33.3)	26.1 (20.7-31.9)	26.0 (21.4-33.3)	.91
Dietary fibre (%E)	2.4 (2.0-2.9)	2.0 (1.7-2.5)[a,b]	2.4 (2.0-2.9)[a,c]	2.7 (2.3-3.2)[b,c]	<.001[a,b] .021[c]	2.1 (2.0-2.8)[a,b]	2.4 (2.0-2.8)[a,c]	2.7 (2.2-3.2)[b,c]	.034[a] <.001[b] .001[c]	2.7 (2.2-3.4)[a,b]	2.4 (2.0-2.9)[a,c]	2.0 (1.7-2.5)[b,c]	<.001[a,b] .001[c]

Alcohol (g)	6.6 (0.0-14.9)	4.1 (0.0-14.2)	7.8 (0.0-19.1)	7.3 (0.0-16.1)	.12	6.8 (0.0-18.9)	7.5 (0.0-18.8)	4.1 (0.0-11.2)	.59	4.9 (0.0-14.3)	5.2 (0.0-14.9)	8.4 (0.0-18.0)	.34
Alcohol (%E)	2.0 (0.0-5.2)	1.3 (0.0-4.6)	2.5 (0.0-6.3)	2.1 (0.0-4.6)	.32	498.8 (291.9-725.6)	430.0 (270.6-680.6)	541.3 (329.2-794.7)	.10	2.1 (0.0-5.5)	1.9 (0.0-4.7)	2.1 (0.0-5.1)	.87
Thiamin (mg)	1.6 (1.2-2.1)	1.6 (1.1-2.0)[a,b]	1.5 (1.2-2.0)[a,c]	1.8 (1.4-2.3)[b,c]	1.00[a] .003[b] .011[c]	1.3 (1.0-1.7)[a,b]	1.5 (1.2-1.9)[a,c]	2.1 (1.6-2.6)[b,c]	.061[a] <.001[b,c]	1.5 (1.1-2.0)[a,b]	1.6 (1.2-2.1)[a,c]	1.7 (1.3-2.3)[b,c]	.28[a] .001[b] .46[c]
Riboflavin (mg)	2.2 (1.6-2.8)	2.1 (1.6-2.8)[a,b]	2.0 (1.5-2.5)[a,c]	2.4 (1.9-3.3)[b,c]	.28[a] <.001[b] .021[c]	1.7 (1.3-2.2)[a,b]	2.1 (1.6-2.6)[a,c]	2.8 (2.3-3.5)[b,c]	<.001[a,b,c]	1.8 (1.4-2.3)[a,b]	2.2 (1.6-2.7)[a,c]	2.5 (2.0-3.3)[b,c]	.002[a] <.001[b] .003[c]
Vitamin C (mg)	103.6 (74.2-149.5)	74.9 (54.2-107.5)[a,b]	102.9 (80.2-144.5)[a,c]	140.5 (102.4-195.8)[b,c]	<.001[a,b,c]	86.5 (58.7-122.2)[a,b]	110.6 (74.8-146.5)[a,c]	130.5 (90.8-183.1)[b,c]	<.001[a,b] .039[c]	128.5 (82.4-177.4)[a,b]	96.2 (71.9-140.3)[a,c]	101.2 (71.5-138.1)[b,c]	.001[a] .004[b] 1.00[c]
Vitamin E (mg)	9.8 (7.0-13.6)	7.9 (5.9-11.2)[a,b]	9.9 (7.1-13.1)[a,c]	11.8 (8.9-15.6)[b,c]	.018[a] <.001[b] .006[c]	9.0 (6.0-13.4)	9.4 (6.9-12.8)	10.5 (8.0-14.3)	.15	9.7 (6.7-13.6)	9.4 (6.6-13.4)	10.2 (7.7-14.0)	.34
Niacin (mg)	50.3 (42.3-61.1)	45.3 (38.2-52.9)[a,b]	48.3 (40.4-56.7)[a,c]	58.7 (49.8-69.4)[b,c]	.17[a] <.001[b,c]	45.1 (37.2-55.3)[a,b]	49.5 (40.8-57.5)[a,c]	56.7 (49.3-68.2)[b,c]	.034[a] <.001[b,c]	44.9 (38.2-53.4)[a,b]	50.3 (42.9-59.5)[a,c]	56.8 (47.6-67.6)[b,c]	.001[a] <.001[b,c]

Folate (ug)	406.7 (303.0-528.2)	358.6 (271.2-500.9)^{a,b}	381.3 (297.1-477.6)^{a,c}	471.7 (369.7-626.5)^{b,c}	1.00^{a} <.001^{b,c}	316.4 (237.1-438.2)^{a,b}	383.2 (303.8-479.3)^{a,c}	503.9 (406.4-644.2)^{b,c}	.001^{a} <.001^{b,c}	376.5 (289.5-512.1)^{a,b}	386.9 (303.3-495.9)^{a,c}	438.7 (321.7-587.7)^{b,c}	1.00^{a} .006^{b} .021^{c}
Vitamin A (ug)	962.6 (739.0-1357.9)	822.4 (589.7-1047.5)^{a,b}	918.9 (725.9-1191.4)^{a,c}	1312.0 (977.6-1699.7)^{b,c}	.009^{a} <.001^{b,c}	808.6 (601.6-1086.6)^{a,b}	960.0 (770.7-1272.5)^{a,c}	1211.5 (925.1-1609.9)^{b,c}	.009^{a} <.001^{b} .006^{c}	892.4 (638.4-1267.7)^{a,b}	967.8 (777.8-1278.7)^{a,c}	1027.8 (840.7-1470.1)^{b,c}	.28^{a} .001^{b} 1.00^{c}
Vitamin D (ug)	4.7 (3.4-6.1)	4.1 (3.1-5.5)^{a,b}	4.7 (3.5-6.1)^{a,c}	5.3 (3.8-6.8)^{b,c}	.034^{a} <.001^{b} .12^{c}	4.5 (3.3-5.7)^{a,b}	4.3 (3.1-5.8)^{a,c}	5.3 (3.9-6.9)^{b,c}	1.00^{a} .001^{b} .003^{c}	4.3 (3.0-5.5)^{a,b}	4.4 (3.4-6.3)^{a,c}	5.2 (3.9-6.5)^{b,c}	1.00^{a} <.001^{b} .021^{c}
Calcium (mg)	795.7 (601.3-1033.7)	761.3 (581.9-993.2)^{a,b}	760.3 (564.4-933.7)^{a,c}	915.3 (642.5-1200.4)^{b,c}	1.00^{a} <.001^{b,c}	633.3 (482.7-814.1)^{a,b}	747.7 (594.8-897.3)^{a,c}	1032.5 (839.6-1278.0)^{b,c}	.009^{a} <.001^{b,c}	681.1 (530.9-913.6)^{a,b}	781.8 (598.7-997.4)^{a,c}	921.7 (716.6-1238.0)^{b,c}	.009^{a} <.001^{b} .001^{c}
Iodine (ug)	109.9 (83.1-146.8)	104.2 (80.4-143.9)^{a,b}	98.3 (75.5-134.9)^{a,c}	127.6 (95.4-170.7)^{b,c}	.62^{a} <.001^{b} .001^{c}	98.5 (71.9-120.5)^{a,b}	101.4 (79.2-134.2)^{a,c}	142.9 (103.2-196.6)^{b,c}	1.00^{a} <.001^{b,c}	93.3 (70.2-124.8)^{a,b}	106.8 (81.0-139.9)^{a,c}	132.6 (102.3-192.1)^{b,c}	.009^{a} <.001^{b} .003^{c}
Iron (mg)	12.7 (10.4-16.1)	11.3 (9.2-14.1)^{a,b}	12.0 (10.2-14.5)^{a,c}	15.4 (12.7-18.4)^{b,c}	.28^{a} <.001^{b,c}	11.1 (9.2-13.2)^{a,b}	12.3 (10.0-15.2)^{a,c}	15.4 (13.0-18.9)^{b,c}	.061^{a} <.001^{b,c}	11.7 (9.7-15.2)^{a,b}	12.5 (10.2-15.9)^{a,c}	14.1 (11.7-17.3)^{b,c}	.42^{a} .001^{b} .021^{c}

Zinc (mg)	13.3 (11.1-16.6)	12.0 (9.5-14.4)[a,b]	12.8 (10.6-15.5)[a,c]	15.8 (13.4-18.7)[b,c]	.28[a] <.001[b,c]	12.0 (9.6-14.5)[a,b]	12.9 (10.6-15.6)[a,c]	15.2 (13.3-18.1)[b,c]	.034[a] <.001[b,c]	11.8 (9.6-14.4)[a,b]	13.2 (11.3-16.4)[a,c]	15.5 (13.1-18.7)[b,c]	<.001[a,b,c]
Potassium (mg)	3314.7 (2781.9-3955.5)	2757.2 (2338.9-3320.9)[a,b]	3187.5 (2888.2-3578.3)[b,c]	4016.5 (3539.9-4796.4)[b,c]	<.001[a,b,c]	2795.6 (2381.3-3306.7)	3202.9 (2862.6-3696.6)	3949.4 (3428.1-4645.5)	<.001[a,b,c]	3163.8 (2666.0-3792.3)[a,b]	3182.8 (2746.3-3753.2)[a,c]	3692.9 (3094.4-4362.5)[b,c]	1.00[a] <.001[b,c]
Sodium (mg)	1971.7 (1520.9-2444.5)	1872.6 (1418.6-2326.3)[a,b]	1876.3 (1496.1-2409.4)[a,c]	2108.1 (1710.2-2640.0)[b,c]	1.00[a] .001[b] .011[c]	1878.8 (1321.8-2428.1)[a,b]	1869.7 (1504.0-2354.9)[a,c]	2136.2 (1694.1-2577.4)[b,c]	1.00[a] .006[b,c]	1631.4 (1173.1-2122.1)[a,b]	1897.0 (1547.4-2318.3)[a,c]	2331.1 (1908.5-2772.2)[b,c]	.009[a] <.001[b,c]
Magnesium (mg)	355.7 (283.0-438.1)	301.4 (239.6-392.5)[a,b]	330.3 (280.6-412.3)[a,c]	425.1 (356.7-492.3)[b,c]	.11[a] <.001[b,c]	281.5 (236.9-344.8)[a,b]	338.6 (286.4-407.3)[a,c]	439.7 (385.6-495.3)[b,c]	<.001[a,b,c]	332.6 (260.0-422.9)[a,b]	339.6 (272.2-420.0)[a,c]	387.1 (312.8-454.0)[b,c]	1.00[a] <.001[b] .011[c]
Phosphorus (mg)	1591.3 (1301.0-1915.6)	1433.5 (1115.8-1747.3)[a,b]	1528.8 (1294.3-1766.7)[a,c]	1872.1 (1591.3-2165.5)[b,c]	.17[a] <.001[b,c]	1367.0 (118.9-1611.7)[a,b]	1529.9 (1287.7-1757.3)[a,c]	1896.6 (1654.1-2206.3)[b,c]	.018[a] <.001[b,c]	1413.0 (1122.6-1679.8)[a,b]	1561.4 (1297.6-1838.7)[a,c]	1858.5 (1560.6-2165.5)[b,c]	.005[a] <.001[b,c]

Food groups													
Other vegetables (serves/d)	1.3 (0.8-2.0)	0.8 (0.5-1.1)[a,b]	1.3 (0.9-1.8)[a,c]	2.3 (1.5-3.2)[b,c]	<.001[a,b,c]	1.1 (0.7-2.0)	1.3 (0.9-2.0)	1.3 (0.9-2.2)	.082	1.3 (0.8-2.1)	1.2 (0.8-1.9)	1.3 (0.8-2.0)	.36
Dark green vegetables (serves/d)	0.2 (0.1-0.4)	0.1 (0.-0.2)[a,b]	0.2 (0.1-0.4)[a,c]	0.5 (0.2-0.7)[b,c]	<.001[a,b,c]	0.2 (0.0-0.4)	0.2 (0.1-0.5)	0.2 (0.1-0.5)	.19	0.3 (0.1-0.5)	0.2 (0.1-0.4)	0.2 (0.0-0.3)	.021†
Red orange vegetables (serves/d)	0.9 (0.5-1.3)	0.5 (0.3-0.8)[a,b]	0.9 (0.6-1.2)[a,c]	1.2 (0.8-1.9)[b,c]	<.001[a,b,c]	0.7 (0.4-1.1)	0.9 (0.5-1.3)	1.0 (0.6-1.5)	.030†	0.9 (0.5-1.5)[a,b]	0.9 (0.6-1.2)[a,c]	0.7 (0.4-1.2)[b,c]	1.00[a]<br>.015[b]<br>.091[c]
Legumes (serves/d)	0.1 (0.0-0.3)	0.0 (0.0-0.2)[a,b]	0.1 (0.0-0.2)[a,c]	0.1 (0.0-0.4)[b,c]	.28[a]<br>.001[b]<br>.19[c]	0.1 (0.0-0.4)	0.0 (0.0-0.2)	0.1 (0.0-0.3)	.35	0.1 (0.0-0.3)	0.1 (0.0-0.3)	0.1 (0.0-0.3)	.96
Seafood (serves/d)	0.3 (0.2-0.5)	0.2 (0.1-0.4)[a,b]	0.3 (0.2-0.5)[a,c]	0.4 (0.2-0.6)[b,c]	<.001[a,b]<br>.19[c]	0.3 (0.2-0.5)	0.3 (0.1-0.4)	0.4 (0.2-0.5)	.016†	0.4 (0.2-0.5)[a,b]	0.3 (0.1-0.5)[a,c]	0.3 (0.2-0.5)[b,c]	.034[a]<br>.008[b]<br>1.00[c]
Red meats (serves/d)	1.1 (0.7-1.6)	0.9 (0.6-1.4)[a,b]	1.1 (0.8-1.5)[a,c]	1.3 (0.9-2.0)[b,c]	.018[a]<br>.001[b]<br>.31[c]	1.2 (0.8-1.8)	1.1 (0.6-1.6)	1.0 (0.8-1.5)	.23	0.9 (0.6-1.3)[a,b]	1.1 (0.8-1.8)[a,c]	1.3 (0.9-1.8)[b,c]	.28[a]<br><.001[b]<br>.31[c]
Wholegrains (serves/d)	1.9 (0.8-3.1)	1.6 (0.8-2.8)	2.0 (0.7-3.2)	2.0 (0.9-3.4)	0.17	0.7 (0.0-1.3)[a,b]	1.9 (1.2-2.7)[a,c]	3.5 (2.6-4.4)[b,c]	<.001[a,b,c]	1.5 (0.8-2.9)	2.0 (1.0-3.2)	2.0 (0.7-3.4)	.20

Refined grains (serves/d)	2.7 (1.7-4.2)	2.7 (1.8-4.0)	2.6 (1.6-4.1)	2.8 (1.7-4.9)	.53	4.2 (2.7-6.0)[a,b]	2.6 (1.8-3.5)[a,c]	1.8 (1.1-2.9)[b,c]	<.001[a,b,c]	2.2 (1.3-3.9)[a,b]	2.7 (1.9-4.1)[a,c]	3.2 (2.0-4.6)[b,c]	.62[a] .021[b] .039[c]
Soy products (serves/d)	0.0 (0.0-0.0)	0.0 (0.0-0.0)[a,b]	0.0 (0.0-0.0)[a,c]	0.0 (0.0-0.0)[b,c]	.096[a] .011[b] 1.00[c]	0.0 (0.0-0.0)[a,b]	0.0 (0.0-0.0)[a,c]	0.0 (0.0-0.0)[b,c]	.35[a] .019[b] .61[c]	0.0 (0.0-0.0)[a,b]	0.0 (0.0-0.0)[a,c]	0.0 (0.0-0.0)[b,c]	1.00[a] .034[b,c]
Milk (serves/d)	1.0 (0.6-1.7)	1.1 (0.7-1.9)[a,b]	0.9 (0.4-1.4)[a,c]	1.1 (0.6-1.9)[b,c]	.034[a] 1.00[b] .31[c]	0.8 (0.3-1.2)[a,b]	1.0 (0.6-1.5)[a,c]	1.6 (1.0-2.4)[b,c]	.11[a] <.001[b,c]	0.8 (0.3-1.2)[a,b]	1.0 (0.6-1.6)[a,c]	1.5 (0.9-2.1)[b,c]	<.001[b,c] .061[a]
Other fruits (serves/d)	1.4 (0.8-2.1)	1.0 (0.5-1.9)[a,b]	1.5 (0.8-2.2)[a,c]	1.6 (1.0-2.3)[b,c]	.001[a,b] 1.00[c]	0.9 (0.5-1.6)[a,b]	1.4 (0.8-2.0)[a,c]	1.9 (1.1-2.8)[b,c]	.001[a] <.001[b] .006[c]	1.6 (0.9-2.4)[a,b]	1.3 (0.7-2.1)[a,c]	1.2 (0.8-2.1)[b,c]	.11[a] .029[b] 1.00[c]
Yoghurt (serves/d)	0.0 (0.0-0.1)	0.0 (0.0-0.0)[a,b]	0.0 (0.0-0.1)[a,c]	0.0 (0.0-0.3)[b,c]	.35[a] <.001[b] .055[c]	0.0 (0.0-0.0)[a,b]	0.0 (0.0-0.1)[a,c]	0.0 (0.0-0.4)[b,c]	<.001[a,b] .007[c]	0.0 (0.0-0.1)	0.0 (0.0-0.1)	0.0 (0.0-0.1)	.68
Nuts and seeds (serves/d)	0.2 (0.0-0.6)	0.2 (0.0-0.5)[a,b]	0.1 (0.0-0.6)[a,c]	0.3 (0.0-0.8)[b,c]	1.00[a] .011[b] .039[c]	0.0 (0.0-0.4)[a,b]	0.2 (0.0-0.7)[a,c]	0.3 (0.0-0.9)[b,c]	.002[a] <.001[b] .12[c]	0.2 (0.0-0.6)	0.2 (0.0-0.6)	0.2 (0.0-0.7)	.86
Fruit juice (serves/d)	0.0 (0.0-0.0)	0.0 (0.0-0.0)	0.0 (0.0-0.0)	0.0 (0.0-0.0)	.16	0.0 (0.0-0.0)	0.0 (0.0-0.0)	0.0 (0.0-0.0)	.49	0.0 (0.0-0.0)	0.0 (0.0-0.0)	0.0 (0.0-0.0)	.26

Discretionary (serves/d)	17.7 (12.4-23.9)	18.9 (13.1-24.7)	16.3 (12.1-22.5)	17.5 (11.6-24.2)	.068	15.4 (11.4-21.8)[a,b]	17.1 (12.4-22.6)[a,c]	19.5 (14.2-27.6)[b,c]	.28[a] .001[b] .070[c]	11.4 (8.2-16.0)[a,b]	17.5 (13.8-21.2)[a,c]	24.8 (20.1-30.1)[b,c]	<.001[a,b,c]
Milk alternatives (serves/d)	0.0 (0.0-0.0)	0.0 (0.0-0.0)	0.0 (0.0-0.0)	0.0 (0.0-0.0)	.22	0.0 (0.0-0.0)	0.0 (0.0-0.0)	0.0 (0.0-0.0)	.55	0.0 (0.0-0.0)[a,b]	0.0 (0.0-0.0)[a,c]	0.0 (0.0-0.0)[b,c]	.001[a] <.001[b] .95[c]
Starchy vegetables (serves/d)	0.6 (0.3-1.0)	0.4 (0.2-0.8)[a,b]	0.6 (0.3-1.0)[a,c]	0.8 (0.4-1.4)[b,c]	.061[a] <.001[b] .001[c]	0.5 (0.2-0.8)[a,b]	0.6 (0.3-1.1)[a,c]	0.7 (0.4-1.1)[b,c]	.42[a] .003[b] .12[c]	0.3 (0.1-0.6)[a,b]	0.6 (0.3-1.0)[a,c]	0.9 (0.5-1.6)[b,c]	<.001[a,b,c]
Processed meats (serves/d)	0.1 (0.0-0.2)	0.1 (0.0-0.2)	0.1 (0.0-0.3)	0.1 (0.0-0.2)	.24	0.1 (0.0-0.2)	0.1 (0.0-0.3)	0.1 (0.0-0.3)	.11	0.0 (0.0-0.1)[a,b]	0.1 (0.0-0.2)[a,c]	0.2 (0.1-0.4)[b,c]	<.001[a,b] .001[c]
Citrus, melons and berries (serves/d)	0.2 (0.0-0.6)	0.2 (0.0-0.6)	0.3 (0.1-0.7)	0.2 (0.0-0.6)	.62	0.2 (0.0-0.4)[a,b]	0.2 (0.1-0.5)[a,c]	0.4 (0.1-0.9)[b,c]	.17[a] .004[b] .07[c]	0.4 (0.1-1.0)[a,b]	0.2 (0.1-0.6)[a,c]	0.2 (0.0-0.3)[b,c]	.005[a] <.001[b] .15[c]
Eggs (serves/d)	0.2 (0.1-0.3)	0.1 (0.0-0.2)[a,b]	0.1 (0.1-0.3)[a,c]	0.2 (0.1-0.3)[b,c]	.13[a] <.001[b] .001[c]	0.1 (0.1-0.3)	0.1 (0.1-0.3)	0.2 (0.1-0.3)	.47	0.1 (0.0-0.2)	0.2 (0.1-0.3)	0.2 (0.1-0.4)	.051
Cheese (serves/d)	0.3 (0.1-0.6)	0.3 (0.1-0.5)	0.4 (0.1-0.6)	0.3 (0.1-.6)	.51	0.3 (0.1-0.6)	0.3 (0.1-0.6)	0.4 (0.1-0.6)	.73	0.2 (0.0-0.5)[a,b]	0.3 (0.1-0.6)[a,c]	0.4 (0.2-0.7)[b,c]	.034[a] <.001[b] .68[c]

Organ meats (serves/d)	0.0 (0.0-0.0)	0.0 (0.0-0.0)	0.0 (0.0-0.0)	0.0 (0.0-0.0)	.14	0.0 (0.0-0.0)	0.0 (0.0-0.0)	0.0 (0.0-0.0)	.36	0.0 (0.0-0.0)[a,b]	0.0 (0.0-0.0)[a,c]	0.0 (0.0-0.0)[b,c]	.94[a] .030[b] .23[c]
Poultry (serves/d)	0.3 (0.2-0.6)	0.3 (0.1-0.4)[a,b]	0.4 (0.2-0.5)[a,c]	0.4 (0.2-0.7)[b,c]	.018[a] <.001[b] 0.31[c]	0.3 (0.2-0.5)	0.4 (0.2-0.6)	0.3 (0.2-0.6)	.26	0.3 (0.2-0.6)	0.3 (0.2-0.6)	0.3 (0.2-0.5)	.99

[1]P values were obtained using the median test and Bonferroni correction for multiple tests to compare all dietary pattern score tertile groups for differences in median values of continuous variables. Differences between groups are denoted by each letter a, b, or c. [†]No differences between groups were observed after Bonferroni correction for multiple tests.

Supplementary Table 3. Number (%) of events of major adverse cardiovascular events (MACE) and individual endpoints of MACE stratified by tertiles of dietary pattern scores (n = 539)

**Dietary pattern**	Bottom tertile (n = 180)	Middle tertile (n = 180)	Top tertile (n = 179)
Factor 1: 'Vegetables-legumes-seafood'[a]			
Five-point MACE	73 (40.6)	47 (26.1)	48 (26.8)
Four-point MACE excluding all-cause mortality	24 (25.6)	26 (14.4)	24 (13.4)
All-cause mortality	47 (26.7)	31 (17.2)	33 (18.4)
Myocardial infarction	9 (5.0)	6 (3.3)	5 (2.8)
Congestive cardiac failure	34 (18.9)	11 (6.1)	16 (8.9)
Ischaemic stroke	6 (3.3)	9 (5.0)	2 (1.1)
Coronary revascularisation	6 (3.3)	3 (1.7)	6 (3.4)
Factor 2: 'Wholegrains-milk-other fruits'[b]			
Five-point MACE	45 (25.0)	64 (35.6)	59 (33.0)
Four-point MACE excluding all-cause mortality	26 (14.4)	39 (21.7)	31 (17.3)
All-cause mortality	30 (16.7)	43 (23.9)	38 (21.2)
Myocardial infarction	6 (3.3)	5 (2.8)	9 (5.0)
Congestive cardiac failure	15 (8.3)	28 (15.6)	18 (10.1)
Ischaemic stroke	6 (3.3)	7 (3.9)	4 (2.2)
Coronary revascularisation	2 (1.1)	6 (3.3)	7 (3.9)
Factor 3: 'Discretionary-starchy vegetables-processed meats'[c]			
Five-point MACE	50 (27.8)	52 (28.9)	66 (36.9)
Four-point MACE excluding all-cause mortality	37 (20.6)	28 (15.6)	31 (17.3)
All-cause mortality	27 (15.0)	33 (18.3)	51 (28.5)
Myocardial infarction	6 (3.3)	7 (3.9)	7 (3.9)
Congestive cardiac failure	21 (11.7)	19 (10.6)	21 (11.7)
Ischaemic stroke	9 (5.0)	8 (4.4)	0 (0.0)

Coronary revascularisation	4 (2.2)	4 (2.2)	7 (3.9)

[a] Bottom tertile ≤-0.48, n = 180; middle tertile -0.47-0.21, n = 180; top tertile ≥0.22, n = 179

[b] Bottom tertile ≤-0.41, n = 180; middle tertile -0.40-0.40, n = 180; top tertile ≥0.41, n = 179

[c] Bottom tertile ≤-0.38, n = 180; middle tertile -0.37-0.40, n = 180; top tertile ≥0.41, n = 179

Supplementary Table 4. Predictors of five-point MACE in univariate analysis unadjusted using Cox regression presented as hazard ratios (95% CI) (n=539)

Risk factor	Hazard ratios (95% CI)
Age	1.11 (1.08, 1.14) P <.001
BMI (kg/m^2) (n = 533)	0.96 (0.92, 0.99) P = .012
Country of birth	
Australia (reference)	1
Greece/ Italy	0.68 (0.46, 1.02) P = .061
Other	0.88 (0.61, 1.28) P = .51
Source of income (n = 538)	
Age Pension only (reference)	1
Other	0.78 (0.57, 1.05) P = .11
Marital status (n = 536)	
Married/de facto (reference)	1
Not married/divorced /separated/widowed /never married/other	1.39 (0.99, 1.96) P = .055
Cigarette smoking status (n = 536)	
Nonsmoker (reference)	1
Ex-smoker	0.90 (0.66, 1.22) P = .49
Current smoker	1.32 (0.64, 2.74) P = .46
Supplement use (vitamins, minerals and/or fish oil)	
No (reference)	1
Yes	1.05 (0.72, 1.53) P = .81
Energy intake	1.00 (1.00,1.00) P = .61
Haemoglobin (g/L) (n = 523)	0.98 (0.97, 0.99) P <.001
Anaemia (n = 523) (Haemoglobin<130g/L)	
No (reference)	1
Yes	1.65 (1.13, 2.41) P = .009

Number of cardiovascular medications	1.13 (1.02, 1.26) P = .019
Frailty status (n = 534)	
Robust (reference)	1
Pre-frail	1.59 (1.14, 2.20) P = .006
Frail	5.47 (3.19, 9.38) P <.001
History of cancer	
No (reference)	1
Yes	1.89 (1.19, 2.98) P = .007
Chronic Kidney Disease (eGFR <60mL/min/1.73m^2) (n = 525)	
No (reference)	1
Yes	1.50 (1.10, 2.06) P = .011

Supplementary Table 5. Associations between dietary pattern factor scores and individual endpoints of MACE using Cox regression presented as hazard ratios (95% CI) (n = 539)

**Dietary Pattern Factor Scores**	Bottom tertile (reference category)	Middle tertile	Top tertile	As continuous variable (per 1 increment)
Factor 1: ‘Vegetables-legumes-seafood’[a]				
**All-cause mortality**				
Model 1	1	0.63 (0.40, 1.00) P = .048	0.66 (0.42, 1.03) P = .067	0.78 (0.62, 0.98) P = .031
Model 2	1	0.79 (0.49, 1.27) P = .33	0.76 (0.47, 1.22) P = .26	0.85 (0.67, 1.07) P = .17
Model 3	1	0.75 (0.46, 1.22) P = .25	0.86 (0.52, 1.40) P = .53	0.88 (0.70, 1.12) P = .30
**Congestive cardiac failure**				
Model 1	1	0.29 (0.15, 0.58) P <.001	0.43 (0.23, 0.77) P = .005	0.61 (0.43, 0.88) P = .006
Model 2	1	0.30 (0.15, 0.60) P = .001	0.49 (0.26, 0.95) P = .035	0.62 (0.42, 0.93) P = .019
Model 3	1	0.31 (0.15, 0.65) P = .002	0.58 (0.29, 1.16) P = .12	0.69 (0.46, 1.03) P = .071
**Coronary revascularisation**				
Model 1	1	0.47 (0.12, 1.89) P = .29	0.95 (0.31, 2.95) P = .93	1.03 (0.64, 1.67) P = .89
Model 2	1	0.43 (0.10, 1.80) P = .25	0.93 (0.27, 3.20) P = .90	0.98 (0.59, 1.61) P = .93
Model 3[†]	1	0.52 (0.12, 2.28) P = .39	1.21 (0.34, 4.34) P = .78	1.07 (0.67, 1.71) P = .80

**Myocardial infarction**				
Model 1	1	0.63 (0.22, 1.76)	0.51 (0.17, 1.53)	0.93 (0.58, 1.49)
		P = .38	P = .23	P = .77
Model 2		0.60 (0.20, 1.75)	0.58 (0.17, 1.95)	1.01 (0.61, 1.65)
		P = .35	P = .38	P = .98
Model 3	1	0.42 (0.13, 1.37)	0.43 (0.11, 1.65)	0.85 (0.47, 1.54)
		P = .15	P = .22	P = .59
**Ischaemic stroke**				
Model 1	1	1.49 (0.53, 4.18)	0.32 (0.07, 1.59)	0.71 (0.38, 1.31)
		P = .45	P = .16	P = .71
Model 2	1	1.97 (0.66, 5.82)	0.42 (0.08, 2.22)	0.85 (0.44, 1.64)
		P = .22	P = .31	P = .63
Model 3	1	2.34 (0.75, 7.32)	0.42 (0.08, 2.39)	0.87 (0.45, 1.67)
		P = .15	P = .33	P = .67
Factor 2: ‘Wholegrains-milk-other fruits’[b]				
**All-cause mortality**				
Model 1	1	1.44 (0.90, 2.29)	1.23 (0.76, 1.98)	1.04 (0.86, 1.26)
		P = .13	P = .40	P = .66
Model 2	1	1.39 (0.84, 2.31)	0.94 (0.54, 1.64)	0.96 (0.79, 1.17)
		P = .20	P = .82	P = .68
Model 3	1	1.69 (1.00, 2.84)	1.11 (0.62, 1.96)	0.99 (0.80, 1.21)
		P = .050	P = .73	P = .90
**Congestive cardiac failure**				
Model 1	1	1.93 (1.03, 3.62)	1.21 (0.61, 2.41)	1.00 (0.78, 1.27)
		P = .040	P = .58	P = .97
Model 2	1	2.05 (1.05, 4.03)	1.38 (0.63, 3.02)	0.99 (0.73, 1.35)
		P = .037	P = .42	P = .96
Model 3	1	2.33 (1.17, 4.65)	1.30 (0.58, 2.93)	0.97 (0.70, 1.36)

		P = .016	P = .53	P = .87
**Coronary revascularisation**				
Model 1	1	3.03 (0.61, 15.02) P = .17	3.47 (0.72, 16.72) P = .12	1.24 (0.72, 2.14) P = .43
Model 2	1	4.39 (0.78, 24.83) P = .095	6.07 (1.00, 36.83) P = .050	1.43 (0.77, 2.67) P = .26
Model 3†	1	4.93 (0.85, 28.76) P = .076	5.68 (0.87, 37.06) P = .070	1.48 (0.76, 2.91) P = .25
**Myocardial infarction**				
Model 1	1	0.83 (0.26, 2.73) P = .76	1.46 (0.52, 4.09) P = .48	0.98 (0.64, 1.52) P = .93
Model 2	1	1.21 (0.34, 4.31) P = .77	2.48 (0.71, 8.66) P = .16	1.12 (0.65, 1.93) P = .69
Model 3	1	0.96 (0.25, 3.65) P = .95	2.54 (0.66, 9.76) P = .17	1.12 (0.62, 2.03) P = .72
**Ischaemic stroke**				
Model 1	1	1.18 (0.40, 3.51) P = .77	0.66 (0.19, 2.35) P = .52	1.02 (0.63, 1.65) P = .93
Model 2	1	1.09 (0.35, 3.46) P = .88	0.91 (0.22, 3.86) P = .90	1.17 (0.64, 2.17) P = .61
Model 3	1	1.26 (0.38, 4.15) P = .71	0.98 (0.23, 4.24) P = .97	1.19 (0.64, 2.21) P = .58
Factor 3: 'Discretionary-starchy vegetables-processed meats'[c]				
**All-cause mortality**				
Model 1	1	1.24 (0.75, 2.07)	1.95 (1.22, 3.11)	1.49 (1.22, 1.81)

		P = .40	P = .005	P <.001
Model 2	1	1.22 (0.71, 2.10)	2.18 (1.25, 3.80)	1.63 (1.27, 2.08)
		P = .47	P = .006	P <.001
Model 3	1	1.25 (0.72, 2.18)	2.26 (1.27, 4.00)	1.63 (1.26, 2.12)
		P = .44	P = .005	P <.001
**Congestive cardiac failure**				
Model 1	1	0.95 (0.51, 1.77)	1.06 (0.58, 1.94)	1.23 (0.95, 1.59)
		P = .87	P = .86	P = .13
Model 2	1	1.04 (0.53, 2.01)	1.24 (0.61, 2.51)	1.40 (1.02, 1.93)
		P = .92	P = .56	P = .040
Model 3	1	1.11 (0.56, 2.21)	1.12 (0.54, 2.34)	1.37 (0.98, 1.91)
		P = .77	P = .77	P = .064
**Coronary revascularisation**				
Model 1	1	1.00 (0.25, 4.00)	1.79 (0.52, 6.12)	1.75 (1.02, 3.00)
		P = 1.00	P = .35	P = .044
Model 2	1	0.87 (0.21, 3.64)	1.52 (0.36, 6.45)	1.73 (0.91, 3.28)
		P = .85	P = .57	P = .096
Model 3[†]	1	0.89 (0.21, 3.74)	1.41 (0.33, 5.97)	1.67 (0.86, 3.23)
		P = .88	P = .64	P = .13
**Myocardial infarction**				
Model 1	1	1.20 (0.40, 3.58)	1.19 (0.40, 3.55)	1.10 (0.70, 1.73)
		P = .74	P = .75	P = .67
Model 2	1	1.59 (0.50, 5.08)	1.63 (0.46, 5.82)	1.29 (0.77, 2.15)
		P = .44	P = .45	P = .34
Model 3	1	1.84 (0.53, 6.34)	1.70 (0.45, 6.41)	1.38 (0.78, 2.43)
		P = .34	P = .44	P = .27
**Ischaemic stroke[#]**				
Model 1	-	-	-	0.77 (0.54, 1.11)

				P = .17
Model 2	-	-	-	0.75 (0.51, 1.09)
				P = .13
Model 3	-	-	-	0.73 (0.50, 1.07)
				P = .11

Notes: Model 1 unadjusted (n = 539 for total, 111 all-cause mortality, 15 coronary revascularisation, 61 congestive cardiac failure, 20 myocardial infarction, and 17 ischaemic stroke); Model 2 adjusted by sociodemographic and lifestyle factors (age (continuous), BMI (continuous), country of birth (Australia v. Greece/Italy v. other), source of income (Age Pension only v. other), marital status (married/de facto v. not married/divorced/separated/widowed/never married/other), smoking status (nonsmoker v. ex-smoker v. current smoker), energy intake (continuous), supplement use including vitamins, minerals, and/or fish oil (yes v. no)) (n = 526 for total, 108 all-cause mortality, 15 coronary revascularisation, 60 congestive cardiac failure, 20 myocardial infarction, and 17 stroke); Model 3 adjusted by Model 2 plus health (haemoglobin (continuous), number of cardiovascular medications (continuous), frailty status (robust v. pre-frail v. frail), history of cancer (yes v. no) and CKD (yes v. no)) (n = 510 for total, 106 all-cause mortality, 57 congestive cardiac failure, 18 myocardial infarction, and 17 stroke).

[†]Due to small numbers frailty status could not be included as covariates for coronary revascularisation. Model 3 (n = 510 for total and 15 coronary revascularisation).

[#]Could not be computed due to low numbers and no stroke events that occurred in a tertile.

[a] Bottom tertile ≤-0.48, n = 180; middle tertile -0.47-0.21, n = 180; top tertile ≥0.22, n = 179

[b] Bottom tertile ≤-0.41, n = 180; middle tertile -0.40-0.40, n = 180; top tertile ≥0.41, n = 179

[c] Bottom tertile ≤-0.38, n = 180; middle tertile -0.37-0.40, n = 180; top tertile ≥0.41, n = 179

Supplementary Table 6. Subgroup analyses by age of associations between dietary pattern factor scores, MACE and individual endpoints of MACE using Cox regression presented as hazard ratios (95% CI) (n = 539)

**Dietary Pattern Factor Scores**	Bottom tertile (reference category)	Middle tertile	Top tertile	As continuous variable (per 1 increment)
Factor 1: 'Vegetables-legumes-seafood'[a]				
**Five-point MACE**				
75-84 years				
Model 1	1	0.78 (0.51, 1.19) P = .25	0.59 (0.37, .94) P = .026	0.84 (0.68, 1.03) P = .093
Model 2	1	0.86 (0.54, 1.37) P = .52	0.80 (0.48, 1.34) P = .40	0.93 (0.75, 1.15) P = .51
>85 years				
Model 1	1	0.30 (0.13, 0.69) P = .005	0.67 (0.37, 1.21) P = .19	0.71 (0.48, 1.05) P = .086
Model 2	1	0.17 (0.07, 0.47) P = .001	0.71 (0.35, 1.44) P = .35	0.69 (0.34, 1.41) P = .31
**Four-point MACE excluding all-cause mortality**				
75-84 years				
Model 1	1	0.73 (0.42, 1.27) P = .27	0.42 (0.22, 0.81) P = .010	0.75 (0.55,1.01) P = .059
Model 2	1	0.77 (0.42, 1.42) P = .40	0.57 (0.27, 1.20) P = .14	0.85 (0.61,1.18) P = .33
>85 years				
Model 1	1	0.20 (0.06, 0.69)	0.65 (0.31, 1.37)	0.65 (0.39,1.07)

		P = .011	P = .26	P = .088
Model 2	1	0.09 (0.02, 0.42)	0.81 (0.34, 1.94)	0.59 (0.30,1.18)
		P = .002	P = .64	P = .14
**All-cause mortality**				
75-84 years				
Model 1	1	0.88 (0.51, 1.52)	0.77 (0.43, 1.35)	0.86 (0.66, 1.10)
		P = .65	P = .36	P = .23
Model 2	1	0.89 (0.49, 1.61)	0.96 (0.51, 1.81)	0.92 (0.72, 1.19)
		P = .70	P = .91	P = .53
>85 years				
Model 1	1	0.35 (0.13, 0.93)	0.58 (0.28, 1.21)	0.64 (0.40, 1.03)
		P = .035	P = .15	P = .064
Model 2	1	0.16 (0.04, 0.58)	0.74 (0.30, 1.83)	0.68 (0.35, 1.34)
		P = .005	P = .15	P = .27
**Congestive cardiac failure**				
75-84 years				
Model 1	1	0.47 (0.22, 1.02)	0.50 (0.23, 1.07)	0.73 (0.49, 1.09)
		P = .058	P = .074	P = .12
Model 2	1	0.51 (0.22, 1.17)	0.56 (0.23, 1.38)	0.75 (0.48, 1.17)
		P = .11	P = .21	P = .20
>85 years				
Model 1	1	0.08 (0.01, 0.61)	0.37 (0.15, 0.96)	0.43 (0.23, 0.82)
		P = .015	P = .040	P = .010
Model 2[ǂ]	1	0.09 (0.01, 0.68)	0.50 (0.19, 1.35)	0.30 (0.11, 0.80)
		P = .019	P = .17	P = .016
**Coronary revascularisation**				
75-84 years	1	0.52 (0.13, 2.19)	0.74 (0.20, 2.74)	1.04 (0.63, 1.72)

		P = .38	P = .65	P = .88
Model 2[*]	1	0.59 (0.14, 2.55)	0.88 (0.21, 3.61)	1.09 (0.65, 1.80)
		P = .48	P = .85	P = .75
>85 years				
Model 1[#]	1	-	-	1.00 (0.22, 4.62)
				P = 1.00
Model 2[#,*]	1	-	-	0.89 (0.13, 6.02)
				P = .90
**Myocardial infarction**				
75-84 years				
Model 1	1	0.69 (0.18, 2.55)	0.35 (0.07, 1.80)	0.94 (0.51, 1.74)
		P = .57	P = .21	P = .85
Model 2[*]	1	0.65 (0.17, 2.48)	0.34 (0.06, 1.95)	0.95 (0.50, 1.83)
		P = .53	P = .23	P = .88
>85 years				
Model 1	1	0.65 (0.12, 3.58)	0.85 (0.19, 3.79)	1.09 (0.45, 2.65)
		P = .62	P = .83	P = .85
Model 2[ǂ,*]	1	0.71 (0.13, 3.96)	1.09 (0.22, 5.44)	0.98 (0.30, 3.23)
		P = .70	P = .92	P = .98
**Ischaemic stroke**				
75-84 years				
Model 1[#]	1	-	-	0.66 (0.30, 1.43)
				P = .29
Model 2[#,*]	1	-	-	0.95 (0.46, 1.95)
				P = .88
>85 years				
Model 1	1	0.50 (0.05, 4.85)	0.81 (0.14, 4.84)	0.93 (0.31, 2.75)
		P = .55	P = .82	P = .89
Model 2[*]	1	0.37 (0.03, 4.10)	0.45 (0.06, 3.59)	0.59 (0.15, 2.26)

		P = .41	P = .45	P = .44
Factor 2: 'Wholegrains-milk-other fruits'[b]				
**Five-point MACE**				
75-84 years				
Model 1	1	1.95 (1.22, 3.13)	1.55 (0.95, 2.53)	1.08 (0.89, 1.30)
		P = .005	P = .082	P = .44
Model 2	1	2.37 (1.41, 3.99)	1.75 (0.97, 3.14)	1.12 (0.89, 1.41)
		P = .001	P = .061	P = .32
>85 years				
Model 1	1	0.72 (0.37, 1.43)	0.91 (0.48, 1.73)	0.97 (0.73, 1.29)
		P = .35	P = .78	P = .83
Model 2	1	0.70 (0.31, 1.59)	0.88 (0.37, 2.08)	0.92 (0.64, 1.34)
		P = .39	P = .77	P = .68
**Four-point MACE excluding all-cause mortality**				
75-84 years				
Model 1	1	2.44 (1.27, 4.68)	1.72 (0.86, 3.44)	1.10 (0.86, 1.42)
		P = .008	P = .12	P = .45
Model 2	1	3.20 (1.60, 6.43)	2.50 (1.13, 5.53)	1.31 (0.94, 1.83)
		P = .001	P = .024	P = .11
>85 years				
Model 1	1	0.66 (0.29, 1.50)	0.66 (0.29, 1.51)	0.86 (0.60, 1.24)
		P = .32	P = .33	P = .42
Model 2	1	0.71 (0.24, 2.07)	0.60 (0.17, 2.10)	0.77 (0.45, 1.34)
		P = .53	P = .43	P = .35
**All-cause mortality**				
75-84 years				
Model 1	1	1.72 (0.97, 3.04)	1.28 (0.70, 2.33)	1.02 (0.81, 1.28)

		P = .064	P = .43	P = .86
Model 2	1	2.20 (1.13, 4.27) P = .020	1.29 (0.61, 2.70) P = .51	1.01 (0.78, 1.30) P = .97
>85 years				
Model 1	1	0.85 (0.37, 1.93) P = .69	1.01 (0.46, 2.22) P = .99	1.06 (0.75, 1.51) P = .74
Model 2	1	0.78 (0.26, 2.31) P = .65	1.34 (0.44, 4.05) P = .61	1.18 (0.74, 1.88) P = .48
**Congestive cardiac failure**				
75-84 years				
Model 1	1	4.04 (1.51, 10.82) P = .005	2.99 (1.08, 8.30) P = .036	1.18 (0.84, 1.65) P = .34
Model 2	1	5.54 (1.93, 15.86) P = .001	3.79 (1.20, 11.96) P = .023	1.30 (0.84, 2.01) P = .23
>85 years				
Model 1	1	0.77 (0.31, 1.91) P = .58	0.30 (0.09, 0.95) P = .041	0.69 (0.45, 1.05) P = .082
Model 2	1	1.28 (0.32, 5.13) P = .73	0.31 (0.04, 2.16) P = .24	0.58 (0.27, 1.21) P = .15
**Coronary revascularisation**				
75-84 years				
Model 1	1	2.60 (0.50, 13.39) P = .25	2.66 (0.52, 13.72) P = .24	1.27 (0.69, 2.33) P = .44
Model 2*	1	2.69 (0.52, 13.94) P = .24	2.68 (0.48, 15.01) P = .26	1.32 (0.67, 2.59) P = .43

>85 years				
Model 1[#]	1	-	-	1.15 (0.31, 4.28)<br>P = .84
Model 2[#,*]	1	-	-	1.14 (0.19, 6.81)<br>P = .88
**Myocardial infarction**				
75-84 years				
Model 1	1	1.43 (0.32, 6.39)<br>P = .64	1.30 (0.29, 5.82)<br>P = .73	0.98 (0.54, 1.76)<br>P = .94
Model 2[*]	1	1.35 (0.30, 6.09)<br>P = .69	1.42 (0.29, 6.84)<br>P = .66	1.00 (0.52, 1.91)<br>P = 1.00
>85 years				
Model 1	1	0.24 (0.03, 2.34)<br>P = .22	1.35 (0.32, 5.65)<br>P = .68	0.90 (0.45, 1.81)<br>P = .76
Model 2[‡,*]	1	0.28 (0.03, 2.82)<br>P = .28	1.70 (0.32, 9.16)<br>P = .54	0.83 (0.35, 1.97)<br>P = .67
**Ischaemic stroke**				
75-84 years				
Model 1	1	1.77 (0.42, 7.40)<br>P = .44	1.02 (0.21, 5.06)<br>P = .98	1.02 (0.57, 1.82)<br>P = .96
Model 2[*]		2.02 (0.47, 8.72)<br>P = .34	2.21 (0.39, 12.41)<br>P = .37	1.50 (0.67, 3.40)<br>P = .33
>85 years				
Model 1	1	0.58 (0.10, 3.49)<br>P = .55	0.28 (0.03, 2.71)<br>P = .27	0.98 (0.41, 2.34)<br>P = .97
Model 2[*]	1	0.58 (0.09, 3.87)<br>P = .57	0.28 (0.02, 3.20)<br>P = .31	1.02 (0.42, 2.48)<br>P = .96
Factor 3: 'Discretionary-starchy vegetables-processed meats'[c]				
**Five-point MACE**				

75-84 years				
Model 1	1	1.24 (0.77, 1.98)<br>P = .38	1.52 (0.97, 2.39)<br>P = .070	1.35 (1.11, 1.64)<br>P = .002
Model 2	1	1.26 (0.76, 2.09)<br>P = .36	1.43 (0.83, 2.45)<br>P = .20	1.36 (1.06, 1.75)<br>P = .017
>85 years				
Model 1	1	0.71 (0.36, 1.41)<br>P = .32	0.96 (0.51, 1.81)<br>P = .90	1.12 (0.85, 1.47)<br>P = .44
Model 2	1	0.76 (0.34, 1.73)<br>P = .51	1.02 (0.45, 2.31)<br>P = .97	1.20 (0.86, 1.69)<br>P = .29
**Four-point MACE excluding all-cause mortality**				
75-84 years				
Model 1	1	0.73 (0.39, 1.35)<br>P = .32	0.91 (0.51, 1.63)<br>P = .75	1.21 (0.93, 1.56)<br>P = .15
Model 2	1	0.79 (0.41, 1.52)<br>P = .48	0.83 (0.41, 1.68)<br>P = .60	1.27 (0.91, 1.76)<br>P = .16
>85 years				
Model 1	1	0.80 (0.35, 1.82)<br>P = .60	0.69 (0.30, 1.59)<br>P = .38	0.97 (0.70, 1.37)<br>P = .88
Model 2	1	1.14 (0.43, 3.03)<br>P = .79	0.71 (0.23, 2.23)<br>P = .56	1.18 (0.76, 1.82)<br>P = .46
**All-cause mortality**				
75-84 years				
Model 1	1	1.58 (0.85, 2.92)<br>P = .15	2.01 (1.11, 3.61)<br>P = .020	1.49 (1.17, 1.89)<br>P = .001
Model 2	1	1.59 (0.82, 3.10)<br>P = .17	1.96 (0.97, 3.96)<br>P = .061	1.59 (1.15, 2.21)<br>P = .006

>85 years				
Model 1	1	0.62 (0.24, 1.58) P = .32	1.69 (0.79, 3.64) P = .18	1.46 (1.02, 2.10) P = .038
Model 2	1	0.35 (0.10, 1.20) P = .096	1.61 (0.56, 4.65) P = .38	1.45 (0.88, 2.39) P = .15
**Congestive cardiac failure**				
75-84 years				
Model 1	1	0.78 (0.35, 1.75) P = .55	1.10 (0.53, 2.31) P = .80	1.31 (0.94, 1.82) P = .11
Model 2	1	0.84 (0.35, 2.00) P = .69	0.84 (0.34, 2.11) P = .71	1.27 (0.82, 1.95) P = .28
>85 years				
Model 1	1	1.05 (0.39, 2.82) P = .94	0.89 (0.31, 2.55) P = .83	1.06 (0.69, 1.61) P = .80
Model 2	1	1.71 (0.47, 6.21) P = .42	1.25 (0.29, 5.47) P = .77	1.36 (0.76, 2.44) P = .30
**Coronary revascularisation**				
75-84 years				
Model 1	1	1.05 (0.21, 5.18) P = .96	2.18 (0.55, 8.71) P = .27	1.95 (1.08, 3.53) P = .027
Model 2*	1	1.07 (0.21, 5.42) P = .94	2.33 (0.48, 11.41) P = .30	2.42 (1.14, 5.11) P = .021
>85 years				
Model 1	1	0.60 (0.04, 9.90) P = .60	0.72 (0.04, 11.63) P = .72	0.99 (0.29, 3.44) P = .99
Model 2*	1	0.65 (0.043, 14.38)	0.25 (0.01, 8.61) P = .44	0.66 (0.18, 2.44) P = .54

		P = .78		
**Myocardial infarction**				
75-84 years				
Model 1	1	0.84 (0.19, 3.74)<br>P = .81	1.04 (0.26, 4.18)<br>P = .95	1.17 (0.64, 2.16)<br>P = .61
Model 2[*]	1	0.93 (0.20, 4.39)<br>P = .93	1.13 (0.23, 5.58)<br>P = .88	1.27 (0.61, 2.66)<br>P = .53
>85 years				
Model 1	1	1.60 (0.29, 8.76)<br>P = .59	1.23 (0.21, 7.34)<br>P = .82	0.97 (0.50, 1.86)<br>P = .92
Model 2[ǂ,*]	1	1.90 (0.34, 10.74)<br>P = .59	1.53 (0.22, 10.38)<br>P = .67	1.03 (0.48, 2.20)<br>P = .95
**Ischaemic stroke**				
75-84 years				
Model 1[#]	1	-	-	0.77 (0.50, 1.20)<br>P = .25
Model 2[#]	1	-	-	0.98 (0.47, 2.07)<br>P = .96
>85 years				
Model 1[#]	1	-	-	0.74 (0.37, 1.46)<br>P = .38
Model 2[#,*]	1	-	-	0.59 (0.28, 1.26)<br>P = .17

Notes: Model 1 unadjusted (for 75-84 years n = 434 for total, 114 five-point MACE, 63 four-point MACE excluding all-cause mortality, 74 all-cause mortality, 12 coronary revascularisation, 38 congestive cardiac failure, 11 myocardial infarction, and 11 ischaemic stroke; for >85 years n = 105 for total, 54 five-point MACE, 33 four-point MACE excluding all-cause mortality, 37 all-

cause mortality, 3 coronary revascularisation, 23 congestive cardiac failure, 9 myocardial infarction, and 6 ischaemic stroke); Model 2 fully adjusted by sociodemographic, lifestyle factors and health (age (continuous), BMI (continuous), country of birth (Australia v. Greece/Italy v. other), source of income (Age Pension only v. other), marital status (married/de facto v. not married/divorced/separated/widowed/never married/other), smoking status (nonsmoker v. ex-smoker v. current smoker), energy intake (continuous), supplement use including vitamins, minerals, and/or fish oil (yes v. no), haemoglobin (continuous), number of cardiovascular medications (continuous), frailty status (robust v. pre-frail v. frail), history of cancer (yes v. no) and CKD (yes v. no)) (for 75-84 years n = 412 for total, 109 five-point MACE, 61 four-point MACE excluding all-cause mortality, 71 all-cause mortality, 36 congestive cardiac failure; for >85 years n = 98 for total, 51 five-point MACE, 31 four-point MACE excluding all-cause mortality, 35 all-cause mortality, and 21 congestive cardiac failure)

[‡]Due to small numbers country of birth, source of income, marital status, smoking status, supplement use, haemoglobin, number of cardiovascular medications, frailty status, history of cancer and CKD could not be included as covariates for coronary revascularisation and myocardial infarction. Model 2 fully adjusted (n = 102 for total and 23 congestive cardiac failure, and n = 103 for total and 9 myocardial infarction).

[*]Due to small numbers country of birth, source of income, marital status, smoking status, supplement use, frailty status, history of cancer and CKD could not be included as covariates for coronary revascularisation, myocardial infarction, and stroke. Model 2 fully adjusted (n = 418 for total and 12 coronary revascularisation, n = 418 for total and 11 stroke, n = 418 for total and 11 myocardial infarction, n = 97 for total and 3 coronary revascularisation, n = 99 for total and 7 myocardial infarction, and n = 99 for total and 6 for stroke).

[#]Could not be computed due to low numbers and/or no events that occurred in a tertile.

For 75-84 years

[a] Bottom tertile ≤-0.48, n = 136; middle tertile -0.47-0.21, n = 153; top tertile ≥0.22, n = 145

[b] Bottom tertile ≤-0.41, n = 147; middle tertile -0.40-0.40, n = 145; top tertile ≥0.41, n = 142

[c] Bottom tertile ≤-0.38, n = 149; middle tertile -0.37-0.40, n = 145; top tertile ≥0.41, n = 140

For >85 years

[a] Bottom tertile ≤-0.48, n = 44; middle tertile -0.47-0.21, n = 27; top tertile ≥0.22, n = 34

[b] Bottom tertile ≤-0.41, n = 33; middle tertile -0.40-0.40, n = 35; top tertile ≥0.41, n = 37

[c] Bottom tertile ≤-0.38, n = 31; middle tertile -0.37-0.40, n = 35; top tertile ≥0.41, n = 39

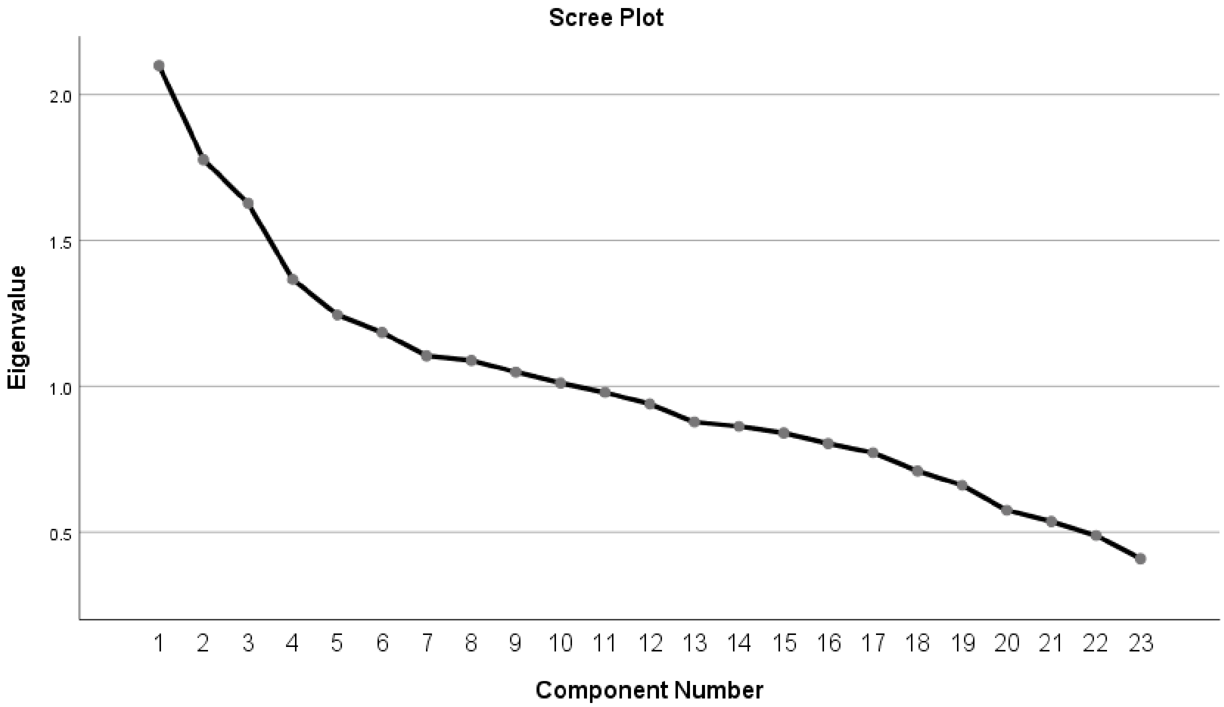

Supplementary Figure 1. Scree plot for the factor analysis

Supplementary Figure 2. Probability of MACE-free survival curves based on dietary pattern score tertiles in unadjusted analyses: (A) all-cause mortality; (B) CCF; (C) coronary revascularisation; (D) MI; (E) stroke. MACE = major adverse cardiovascular event; CCF = congestive cardiac failure; revas = coronary revascularisation; MI = myocardial infarction; Cum = cumulative; Futime = follow-up time.

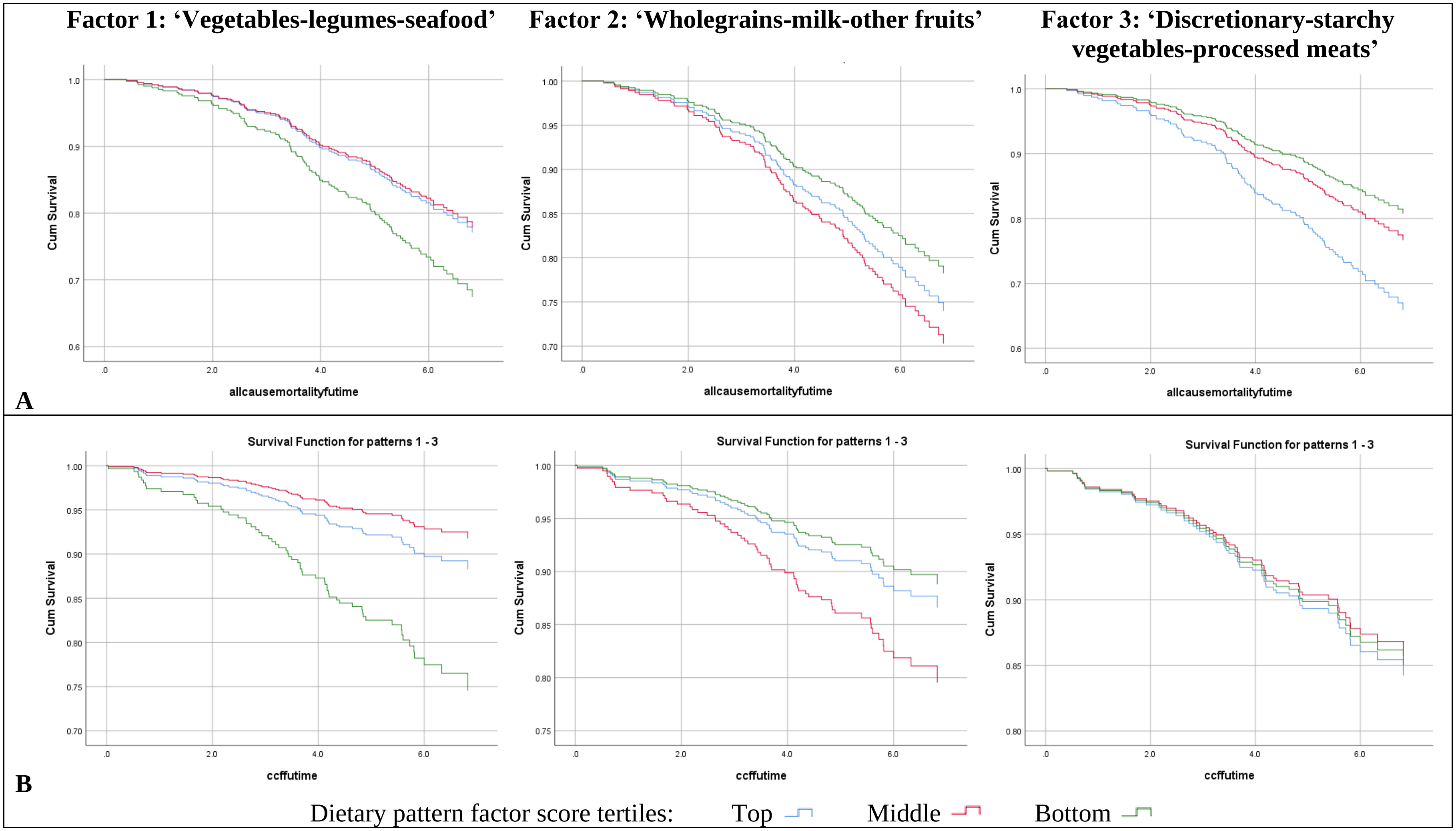

Factor 1: 'Vegetables-legumes-seafood' **Factor 2: 'Wholegrains-milk-other fruits'** **Factor 3: 'Discretionary-starchy vegetables-processed meats'**

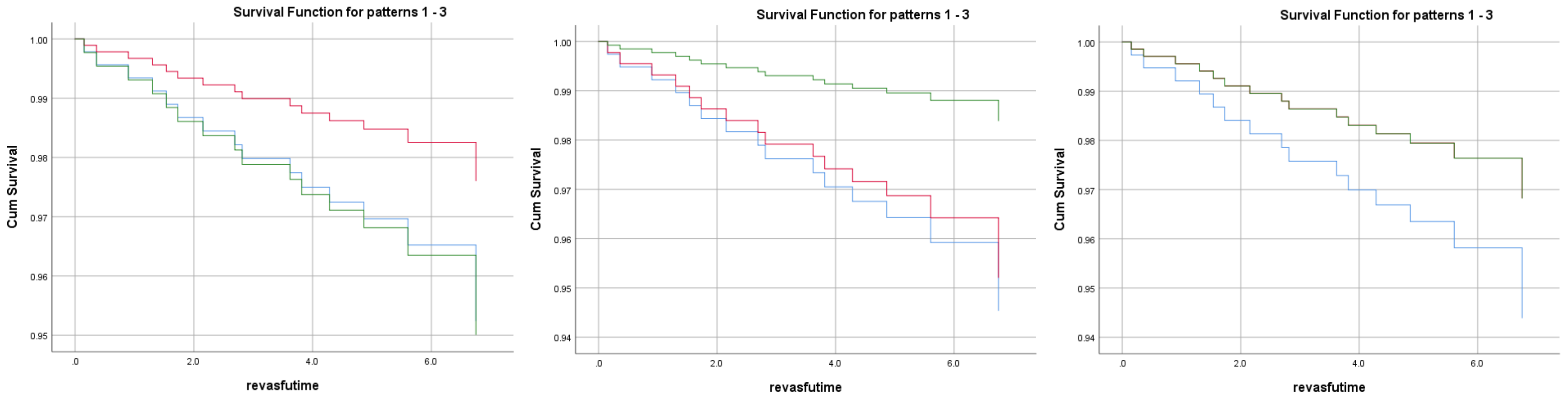

C

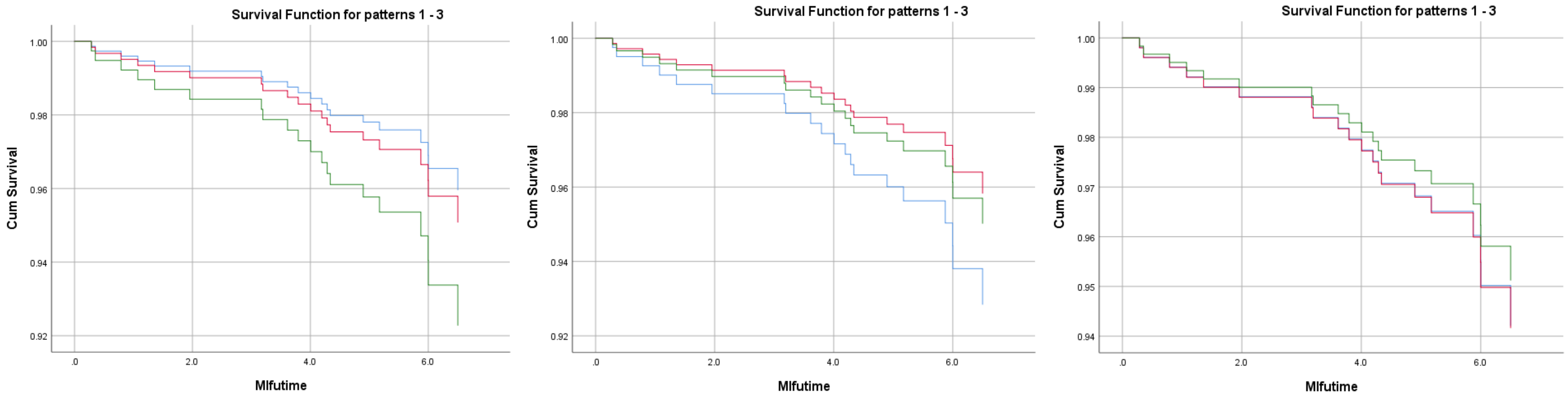

D

Dietary pattern factor score tertiles: Top Middle Bottom

Factor 1: ‘Vegetables-legumes-seafood’ **Factor 2: ‘Wholegrains-milk-other fruits’** **Factor 3: ‘Discretionary-starchy vegetables-processed meats’**

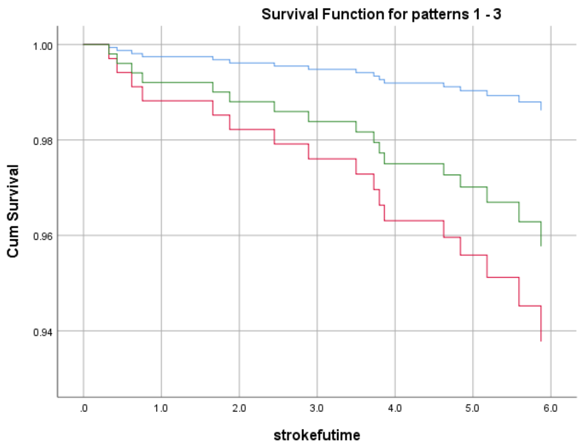

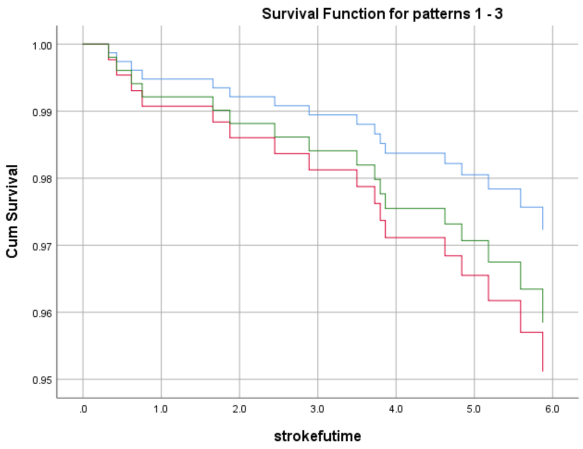

E

Dietary pattern factor score tertiles: Top Middle Bottom

Supplementary Figure 3. Probability of MACE-free survival curves based on dietary pattern score tertiles in fully adjusted analyses: (A) all-cause mortality; (B) CCF; (C) coronary revascularisation; (D) MI; (E) stroke. MACE = major adverse cardiovascular event; CCF = congestive cardiac failure; revas = coronary revascularisation; MI = myocardial infarction; Cum = cumulative; Futime = follow-up time.

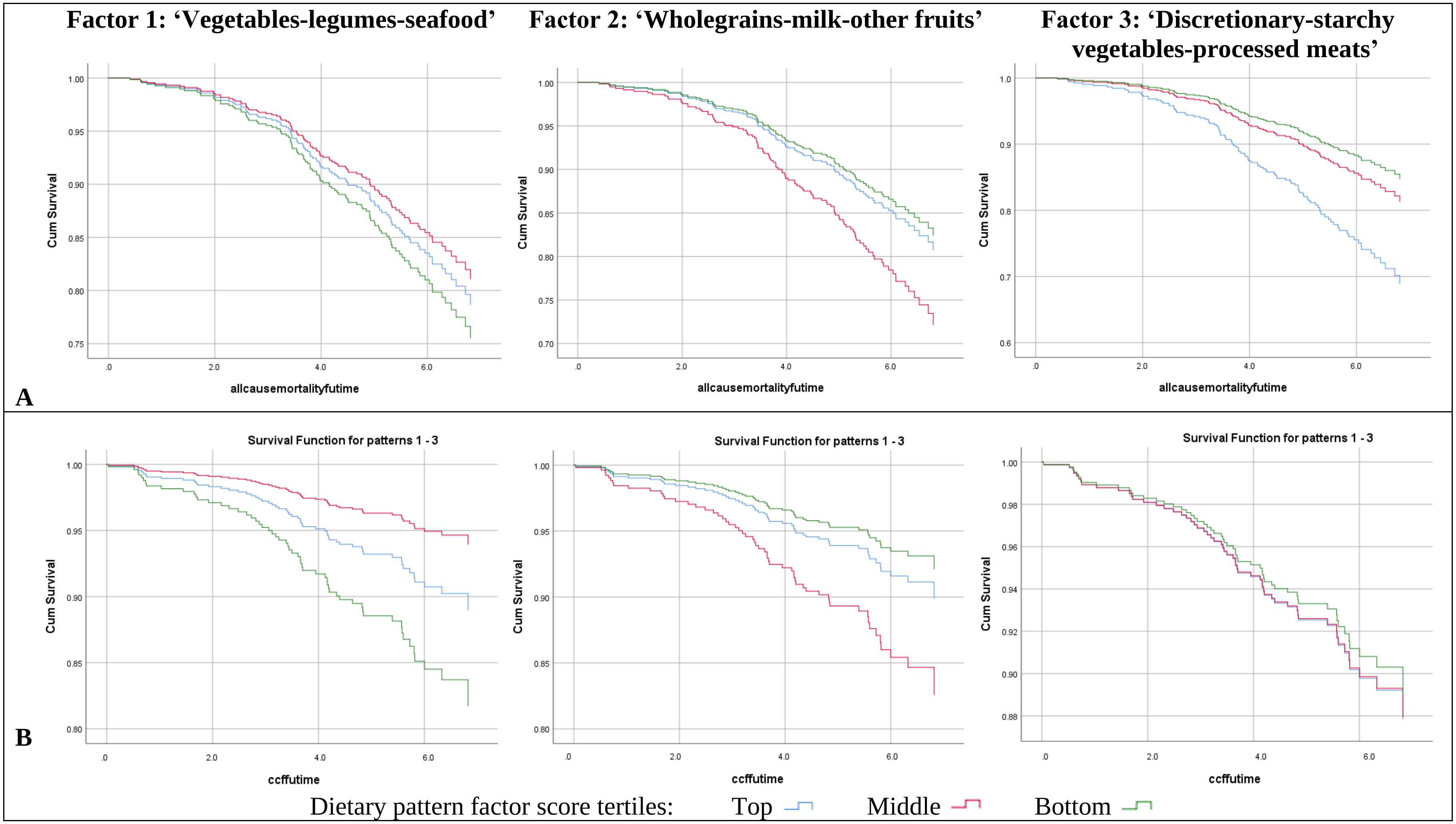

Factor 1: 'Vegetables-legumes-seafood' **Factor 2: 'Wholegrains-milk-other fruits'** **Factor 3: 'Discretionary-starchy vegetables-processed meats'**

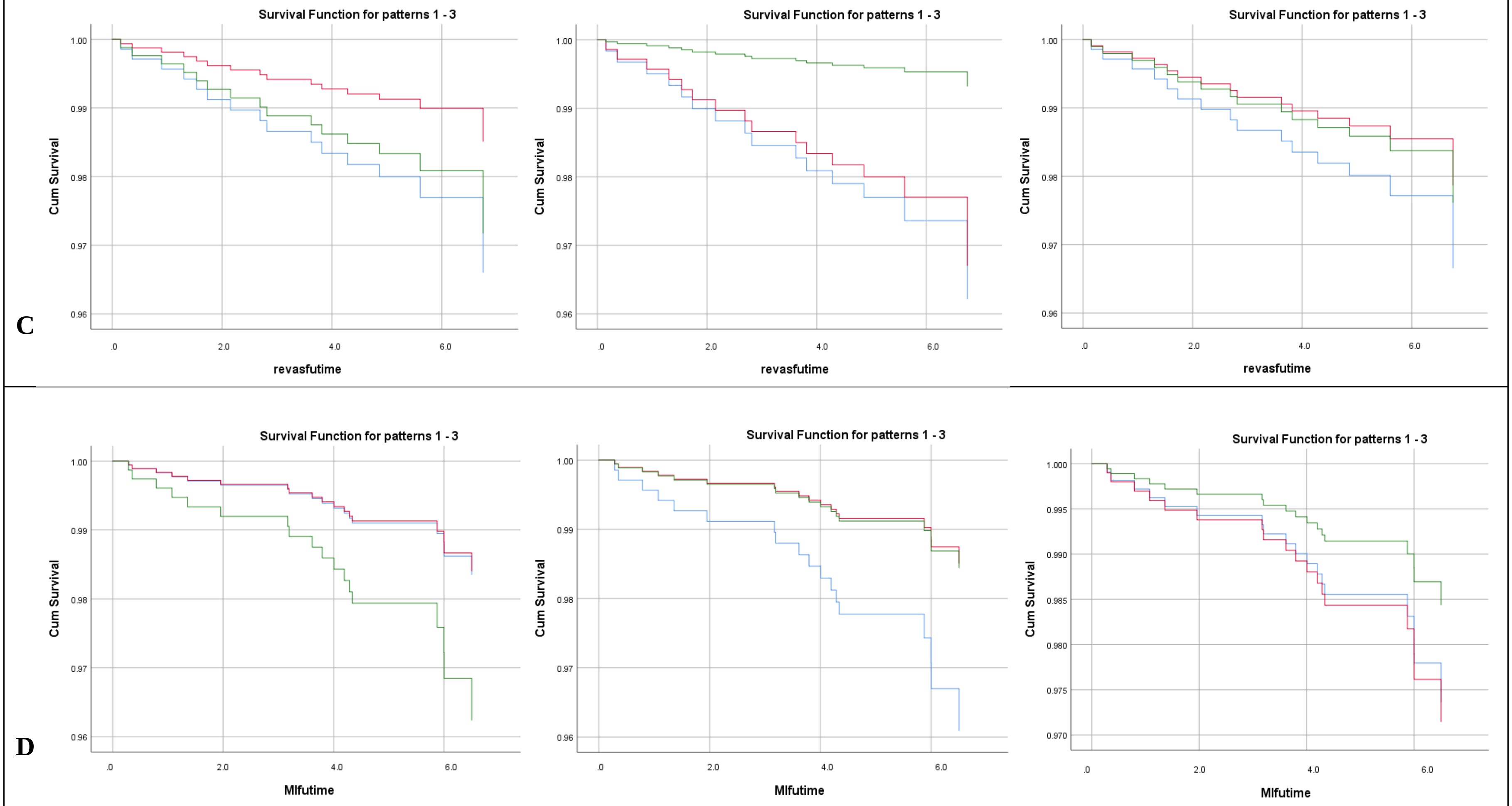

Dietary pattern factor score tertiles: Top Middle Bottom

Factor 1: 'Vegetables-legumes-seafood' **Factor 2: 'Wholegrains-milk-other fruits'** **Factor 3: 'Discretionary-starchy vegetables-processed meats'**

E

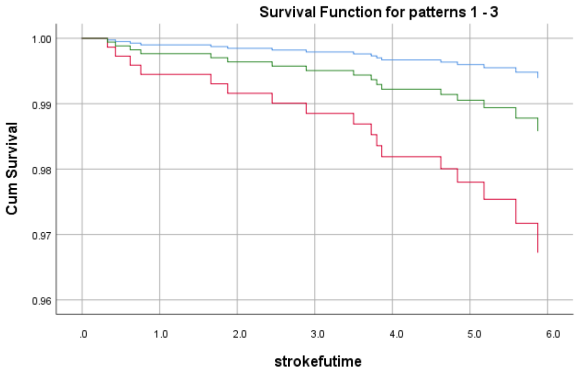

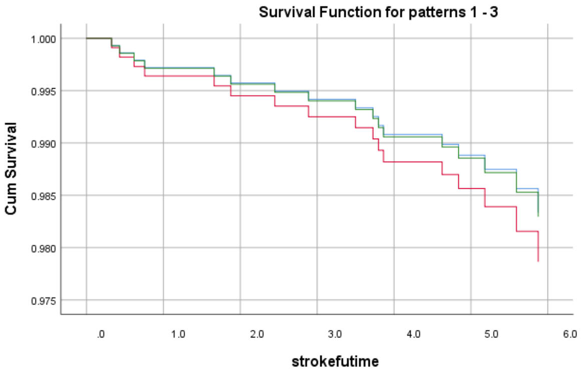

Dietary pattern factor score tertiles: Top Middle Bottom
